# Infrared Spectroscopic Signatures of the Fluorous Effect Arise from a Change of Conformational Dynamics

**DOI:** 10.1021/jacs.4c18434

**Published:** 2025-03-25

**Authors:** R. Cruz, M. R. Becker, J. Kozuch, K. Ataka, R. R. Netz, J. Heberle

**Affiliations:** † Experimental Molecular Biophysics, Freie Universität Berlin, Arnimallee 14, Berlin 14195, Germany; ‡ Theoretical Bio- and Soft Matter Physics, Freie Universität Berlin, Arnimallee 14, Berlin 14195, Germany

## Abstract

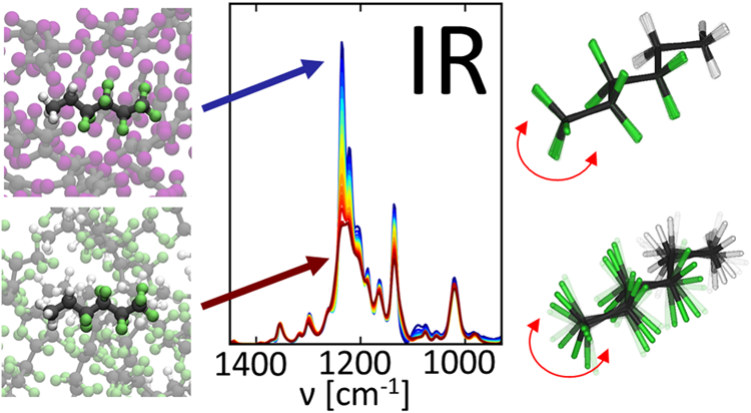

Per- and polyfluoroalkyl substances (PFAS) are synthetic compounds widely employed in society due to their chemical inertness. These substances accumulate in the environment, from where they enter human bodies, leading to harmful effects like cancer. PFAS exhibit omniphobic properties, which often cause them to separate from both aqueous and organic phases, forming a fluorous phase. Yet, the physical nature of this fluorous effect is unknown. Here, we shed light on the fluorous effect by analyzing the infrared absorption spectra of perfluorinated and semifluorinated alkanes in various solvents. We find that specific bands in the C–F stretching vibrational region exhibit selective behaviors in fluorous and nonfluorous environments. In a fluorous environment, these bands undergo significant broadening, and the asymmetric CF_3_ stretching bands decrease in intensity. Using static density functional theory calculations and force-field molecular dynamics simulations, we decipher the underlying molecular mechanisms: The decrease in absorption intensities is related to the intermolecular vibrational coupling of the perfluoroalkyl chains, while an acceleration of conformational changes in the carbon backbone of the molecules causes the observed band broadening. Given the high specificity of the reported spectral changes to a fluorous environment, bands in the C–F stretching range can serve as spectroscopic markers for the fluorous phase, facilitating the study of PFAS aggregation. Such knowledge can lead to the rational design of absorber materials for PFAS, which are aimed at mitigating their environmental impact.

## Introduction

Per- and polyfluoroalkyl substances (PFAS) are organic compounds that contain high degrees of fluorine and are well known to be both hydrophobic and lipophobic (omniphobic).^1^ In multicomponent mixtures, this leads to the formation of a fluorous phase, which separates both from aqueous and organic phases, a phenomenon that is commonly referred to either as the “fluorophilic” or “fluorous” effect.^1^ This unique property of PFAS is utilized in countless applications in biomedicine,^1,2^ catalysis,^3,4^ or interfacial science.^5^ The lipophobic character, for example, facilitates various forms of self-assembly of amphiphilic perfluoroalkyl-alkane diblocks, known as semifluorinated molecules, leading to the formation of stable superstructures such as membranes, vesicles, and micelles,^6,7^ as well as a variety of liquid crystal phases.^8^ Recently, renewed interest in fluorophilic interactions has been sparked, as PFAS have been found to accumulate in the environment and PFAS contamination has been linked to a variety of human diseases.^9,10^ In order to efficiently remove PFAS from the environment, absorbent materials are being developed. One promising avenue is to use different kinds of fluoroalkylated materials in the form of metal–organic frameworks,^11−13^ polymers,^14−17^ gels,^18−20^ etc. The common underlying hypothesis of these works is that fluorophilic interactions cause attraction of PFAS and/or repulsion of nonfluorinated compounds, leading to a high selectivity of the binding of absorber materials toward PFAS. However, a detailed understanding of the underlying mechanisms is still lacking.

On a fundamental level, the fluorous effect is studied in simple model systems, often two-component mixtures, either water-perfluoroalkane or alkane-perfluoroalkane. The increased hydrophobicity of PFAS compared to (nonfluorinated) hydrocarbons has been linked in molecular dynamics (MD) simulations to the large size of fluorine atoms relative to hydrogen, for both bulk and solid–liquid interfaces,^21,22^ which is not sufficiently compensated by fluorine’s dispersive or Coulombic interactions with water. In more complex systems, the hydrophobicity of fluorinated compounds can also be caused by structural changes of fluorinated molecules.^23^ The reason for the lipophobicity of PFAS, on the other hand, remains debated. Early works have claimed that the low polarizability of fluorine compared to hydrogen leads to unusually weak dispersive interactions between H and F atoms.^24,25^ This view has been challenged by high-level quantum chemistry simulations.^26−28^ Additionally, the aggregation of ordered regions with strong dipole–dipole interactions between perfluoroalkyl chains has been suggested as a possible explanation for PFAS lipophobicity.^29^ Recently, it has been found by exhaustive quantum chemical calculations that the perfluoroalkane and alkane geometries are incommensurable with each other, which leads to an arrangement with low intermolecular attraction.^27^ Experimentally, the structure and dynamics of the fluorous phase in binary mixtures have been investigated with the help of various spectroscopic techniques. In perfluoroalkane/alkane mixtures, ^129^Xe nuclear magnetic resonance spectroscopy studies found evidence of nanosegregation,^30^ and Fourier transform infrared spectroscopy (FTIR) showed that alkane molecules preferentially coil up into globular shapes in these mixtures.^31^ Furthermore, the aggregation of Langmuir monolayers of semifluorinated alkanes and lipids at the air–water interface has been studied using surface pressure–surface area isotherms and interpreted with the help of reflection–absorption infrared spectroscopy, indicating a strong chain length dependence of the aggregation process.^32,33^

Infrared (IR) spectroscopy is a sensitive and nondestructive technique that allows for the analysis of the microscopic chemical environment of a molecule via the study of its vibrational dynamics. In this work, we report on the observation of vibrational spectroscopic marker bands of the fluorous phase and explain their origins using theoretical methods. By studying FTIR spectra of semifluorinated and perfluorinated alkanes in a broad range of nonfluorinated solvents, including solvents of different polarity and hydrogen-bonding properties as well as aromatic solvents, we show that the spectral bands assigned to various C–F stretching vibrations are distinctly different in pure PFAS liquids than in solutions of nonfluorous solvents. All considered C–F stretching bands exhibit increased bandwidths in fluorous compared to nonfluorous environments. Additionally, CF_3_ stretching bands display decreased intensity. We explain these results with the help of static density functional theory (DFT) vibrational analysis and force-field-based molecular dynamics (FF-MD) simulations: the decrease in the intensity of CF_3_ stretching bands is caused by intermolecular vibrational coupling between different perfluoroalkyl chains. The increased bandwidths of C–F stretching bands on the other hand can be explained by a common mechanism, namely, faster conformational dynamics of the carbon backbone of semi- and perfluoroalkanes in fluorous liquids compared to nonfluorous solvents. The high selectivity of the reported spectral changes toward a fluorous environment renders the C–F stretching vibrational bands as suitable spectroscopic markers of perfluoroalkyl molecules in the fluorous phase, allowing the investigation of fluorous interactions and the assembly of the fluorous phase.

## Methods

### Compounds and Sample Preparation

**Figure 1:**
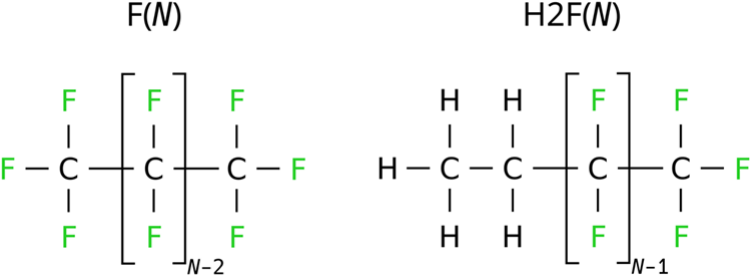
Chemical structures of the studied perfluorinated compounds F(*N*) and semifluorinated compounds H2F(*N*).

We study linear perfluorinated alkanes of different lengths (CF_3_(CF_2_)_*N*−2_CF_3_), which are abbreviated as F(*N*), with *N* being the number of fluorinated carbons, and perfluoroalkyl-alkanes CH_3_CH_2_(CF_2_)_*N*−1_CF_3_, also called semifluorinated compounds, abbreviated as H2F(*N*) (Figure 1). All fluorinated samples were supplied by Synquest Lab with 99% purity. We perform concentration-dependent measurements in tetrachloroethylene (TCE, abcr, 99%), as well as diluted measurements in various other solvents: acetonitrile (Sigma-Aldrich, 99.9%), dodecane (Merck, 99%), ethanol (99.5%, Carl Roth), hexane (EMPARTA, 98.5%), and toluene (Carl Roth, 99.5%).

Spectra of H2F4 in different solvents are recorded at a concentration of 150 mM. When trying to prepare 150 mM solutions of F6, it phase separated in various solvents, forming a fluorous phase that settled at the bottom of the container. Suspensions formed only after vigorous shaking, appearing milky but quickly resolving into two distinct phases within seconds. Instead, a concentration of 10 mM F6 is used, where no such phase separation was observed. For 150 mM H2F4 solutions, no phase separation was observed. The spectra of per- and semifluorinated molecules of different lengths (H2F6, H2F8, H2F10, F8, F10, and F12) in TCE are recorded at a concentration of 10 mM.

All liquid samples are measured under ambient conditions. Solid samples with low melting temperatures (H2F10, F10) are warmed with a heat gun slightly above room temperature (∼40 °C) to keep them liquid while recording the spectra. The spectrum of solid F12 is measured in transmission by using a KBr pellet.

### FTIR Spectroscopy

The extinction coefficient of a sample can be directly measured by normal-incidence transmission FTIR spectroscopy. However, this approach becomes impractical at high concentrations, as the strong absorptivity of fluorinated molecules blocks most infrared radiation, unless extremely thin sample layers are used. This not only complicates sample exchange but also requires precise control of the sample thickness to enable solvent background subtraction. A more practical approach is to record the spectra in the attenuated total reflection (ATR) configuration.

ATR spectra are composed of a linear combination of a transmission-like and a reflection-like component. These are driven by the Im(ϵ(ω)) and  functions of the complex dielectric constant of the sample (ϵ), respectively. The relative increase of the  component at high concentrations results in a red shift of vibrational bands and an intensity increase of low-frequency peaks. As noted by Hasegawa,^32^ this effect is purely optical in nature and must be eliminated before the spectrum can be properly interpreted. The two spectral components are separated using Kramers–Kronig relations^33−35^ and only the transmission-like component (Im(**ϵ**)) is used for the analysis presented in this work. The global phase offset required for ATR configuration is approximated to 90° (see Supporting Information Section S2 for more details). ATR spectra are recorded using a single-reflection silicon crystal from IRUBIS GmbH (Munich, Germany). Spectra are recorded with a Vertex 70v spectrometer from Bruker (Rheinstetten, Germany) at a spectral resolution of 2 cm^–1^.

H2F4 concentration-dependent spectra are measured against a background recorded with pure tetrachloroethylene (TCE) (200 μL) using for both sample and background spectra a total of 512 coadditions. The same procedure is used to record the spectra of the F(*N*) and H2F(*N*) series at low concentrations and in their pure form, except for pure F12 in KBr. Lastly, a background with no sample (air) was used as reference. Spectra of H2F4 and F6 in different solvents are measured against a background spectrum of the pure solvent.

For the parameter estimation of experimental peaks, the frequencies of peak maxima are first determined using a continuous wavelet transform. A second derivative Lorentzian wavelet is used as analyzing function with γ values between 3 and 6 cm^–1^. Spectra are then fitted to a sum of Lorentzian distributions by keeping maxima frequencies fixed and using the intensities and bandwidths as free parameters of the fit (see Section S4 in the Supporting Information).

### DFT Calculations

Static DFT calculations are conducted with the ORCA 5.0.3 software suite^36^ with the B97M-V exchange correlation functional employing def2-TZVP basis sets.^49,50^ All systems besides the single molecules in vacuum are embedded in an implicit solvation model using the conductor-like polarizable continuum model^37^ with a static dielectric constant of ε = 2.5, corresponding to pure liquid TCE. While the polarizable continuum model is considered to work well to describe the influence of polarizability on spectral properties of molecules for weakly polar solvents, it is known that those models break down for highly polar solvents.^38^ Polar solvents usually strongly alter the local charge distribution around solutes, which cannot be accurately described by a continuum model. This is why we only model the polarizability of TCE with a polarizable continuum model and do not employ similar modeling of other solvents that are studied experimentally. More information on the DFT calculations can be found in Section S7 in the Supporting Information.

### MD Simulations

All FF-MD simulations are conducted in GROMACS 2022^39^ using the OPLS/AA force field for TCE^40^ and the OPLS/AA reparametrization for perfluoroalkanes^13^ for F6 simulations. FF simulations for H2F4 presented in the Supporting Information employ a modification of the OPLS/AA force field introduced in ref (22). Note that this force field has not been tested against liquid properties of semifluoroalkyl compounds as of now. Simulations are carried out with a time step of 2 fs. Lennard-Jones interactions are cut off at a distance of 1 nm, and the Lennard-Jones potential is switched to zero at 1.1 nm. Electrostatic interactions are treated with the smooth particle mesh Ewald method,^41^ using a real space cutoff of 1 nm. The temperature is kept constant at *T* = 300 K with a canonical sampling through a velocity rescaling thermostat.^42^ All simulation boxes contain 1000 molecules in total, ranging at various concentrations from 5 F6 and 995 TCE molecules to the pure F6 liquid containing 1000 F6 molecules. After equilibration of 2 ns in the NpT ensemble using the Berendsen barostat^43^ at *p* = 1 bar, sampling is conducted in the NVT ensemble for a minimum of 4 ns (for the pure liquid) and a maximum of 100 ns (for the diluted system). Positions are written out at every time step in order to calculate spectra with a sufficient frequency resolution.

To calculate the absorption spectra, we determine the total dipole moment **M** of the system in every step. The dielectric spectrum χ(ω) at angular frequency ω is calculated using the Green–Kubo relation in conjunction with the Wiener–Khinchin theorem to evaluate autocorrelation functions in the frequency domain
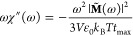
1Here, *V* denotes the simulation box volume, ε_0_ refers to the dielectric permittivity of vacuum, *k*_B_ is the Boltzmann constant, *T* is the absolute temperature, and *t*_max_ is the simulation length. We employ the Fourier transformation convention
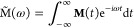
2where *t* is time. The energy absorption is thus proportional to the power spectrum of the time derivative of the total dipole moment ωχ″(ω) ∝ *S*_**ṀṀ**_(ω), where we define the power spectrum of a general vectorial quantity **v** as
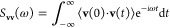
3

### Normal Mode Projections

For projecting MD trajectories onto normal modes, we first determine geometry-optimized reference structures **x**_ref_^±^ with left-handed and right-handed helicity using the same force field as employed in the FF-MD simulations. We then individually align the molecules from FF-MD trajectories, consisting of atoms with positions **x**(*t*), through a translational and rotational fit to the geometry-optimized reference structures by minimizing the mean square distance between the carbon atoms using algorithms implemented in the MDAnalysis software package.^44−46^ Aligning by minimizing the mean square distance between all atoms leads to slightly less robust but overall similar results. In each frame of the MD simulation, the resulting fitted structure **x**_fit_(*t*) is then projected onto vibrational normal modes, by evaluating the scalar product with the normal mode vectors **η**_*k*_^±^. The resulting normal mode trajectories are given by
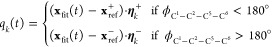
4

## Results and Discussion

### Experimental Results

To address the question of whether fluorous interactions can be detected in the vibrational spectrum of PFAS, we design the following experiment: we measure the FTIR spectrum of polyfluorinated molecules in nonfluorinated solvent at varying concentrations. At low concentrations, most molecules are surrounded by the nonfluorinated solvent, whereas at higher concentrations they will come into contact with each other, thus becoming exposed to a fluorous environment. This approach allows us to study the impact of a fluorous environment on the IR spectrum via recording spectral changes as a function of the concentration of fluorinated species.

**Figure 2:**
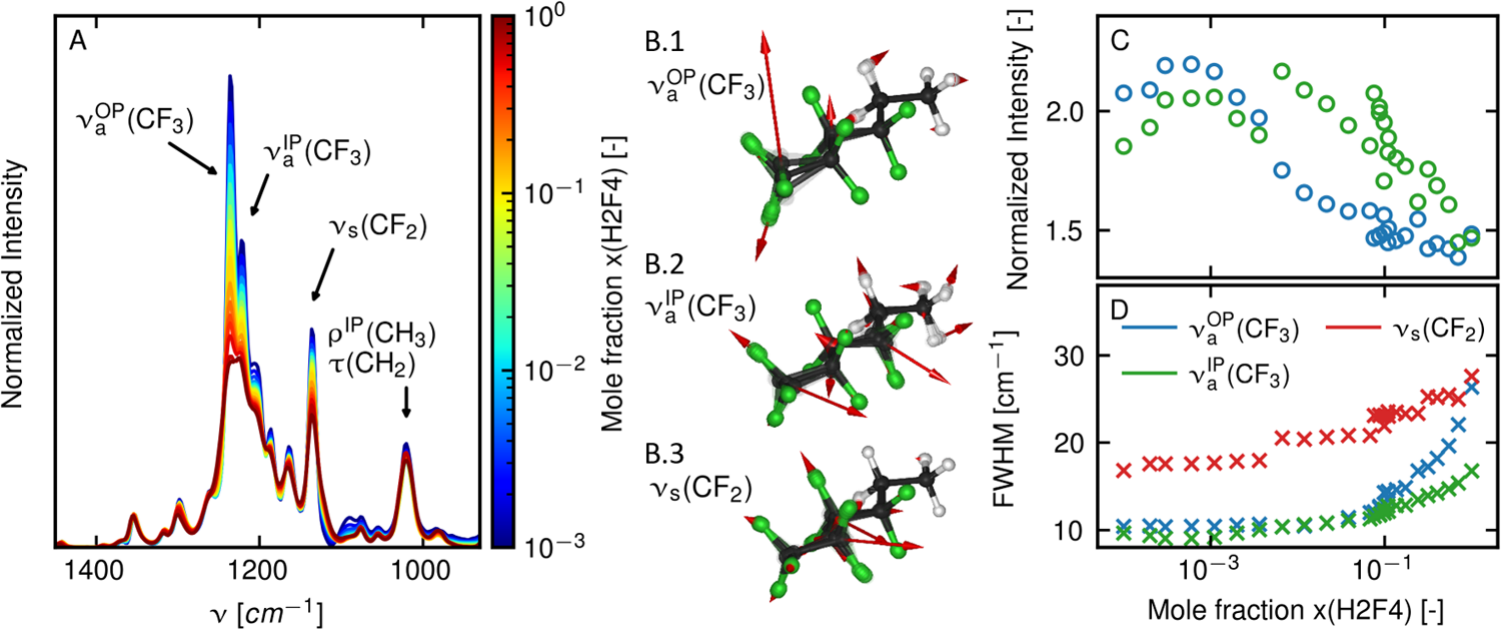
FTIR spectra of H2F4/TCE solutions at varying concentrations of H2F4 ranging from very dilute to pure H2F4 liquid. (A) FTIR spectra with band assignment normalized to the integrated intensity of the ν_s_(CF_2_) band. (B) Visualization of ν_a_^OP^(CF_3_), ν_a_^IP^(CF_3_), and ν_s_(CF_2_) normal modes with atomic displacements shown as arrows. (C) Peak areas of ν_a_^OP^(CF_3_) (blue circles) and ν_a_^IP^(CF_3_) (green circles) normalized with respect to ν_s_(CF_2_) for different molar ratios of H2F4. (D) Bandwidths of ν_a_^OP^(CF_3_), ν_a_^IP^(CF_3_), and ν_s_(CF_2_) bands at different mole ratios.

First, we record FTIR spectra of H2F4 in tetrachloroethylene (TCE) at concentrations ranging from a mole fraction of *x* = 10^–4^ to pure H2F4 (Figure 2A). We choose this solvent because it is transparent in the whole mid-IR region between 930 and 4200 cm^–1^ and because H2F4 is fully miscible with this solvent in the entire concentration range. The resulting spectra (Figure 2A) are normalized with respect to the peak at 1135 cm^–1^. Most notably, they reveal a strong dependence of the dominant absorption peaks on the concentration of H2F4.

**Figure 3:**
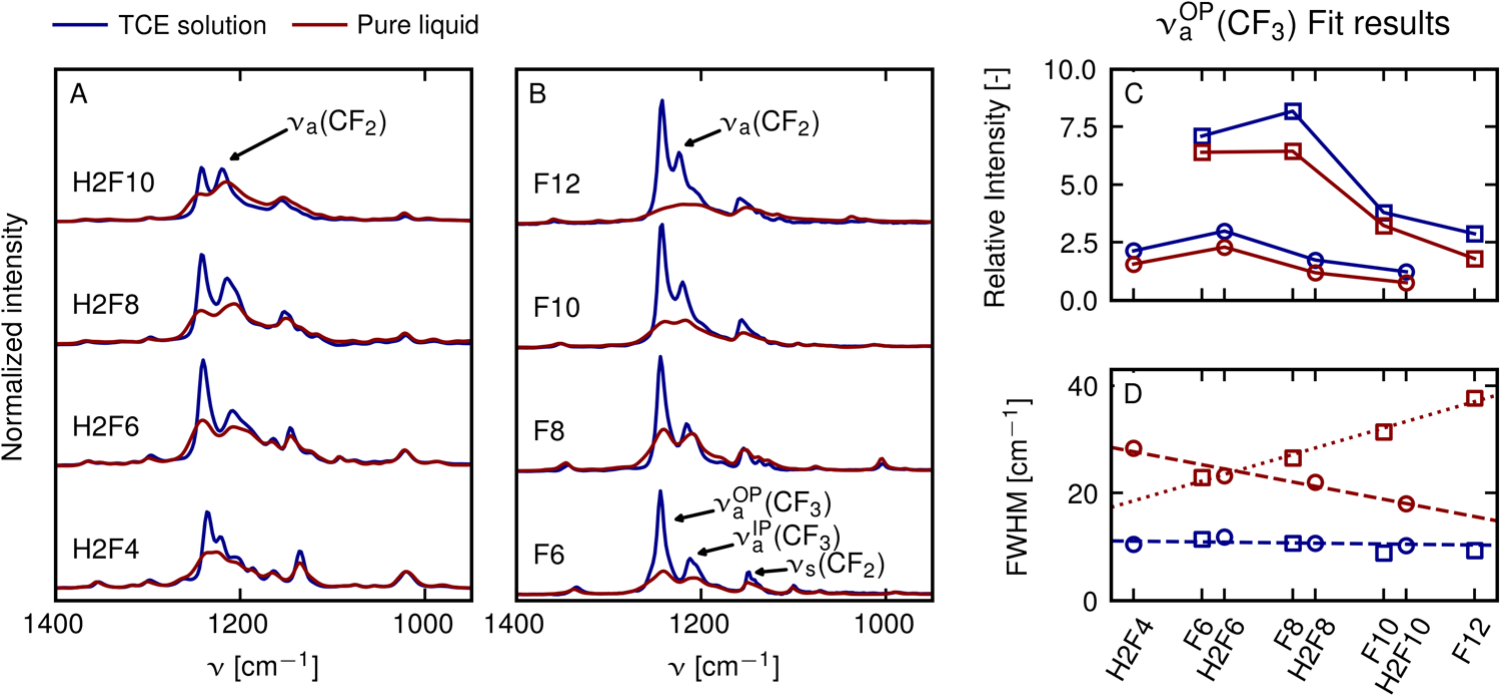
(A) FTIR spectra of pure H2F(*N*) (red lines) and 10 mM H2F(*N*) in TCE solution (blue lines), normalized to the area of ν_s_(CF_2_) peak for *N* = 4, 6, 8, and 10. (B) FTIR spectra of the fully fluorinated analogues F(*N*) under the same conditions for different molecular lengths *N* = 6, 8, 10, 12. (C) Comparison of ν_a_^OP^(CF_3_) normalized peak areas in TCE solution (blue lines) versus pure samples (red lines) of perfluorinated molecules of different lengths. (D) Line widths, quantified by the full width at half-maximum (FWHM), in TCE solution (blue lines) and pure samples (red lines) of the ν_a_^OP^(CF_3_) band for the same molecules.

We assign absorption bands to molecular vibrations by comparing the FTIR spectrum with the DFT-based normal-mode analysis of single molecules in vacuum. The IR spectrum of H2F4 is characterized by a complex overlap of various C–F stretching (ν(CF_*n*_)) bands (Figure 2A). At low concentrations, the spectrum is dominated by a band at 1236 cm^–1^, which is assigned to the out-of-plane asymmetric stretching of the terminal CF_3_ group (ν_a_^OP^(CF_3_), Figure 2B). The peak height of this band after normalization decreases with increasing H2F4 concentration, such that, in the pure sample, it equals the second strongest band at 1220 cm^–1^, which is assigned to in-plane asymmetric stretching of the CF_3_ group (ν_a_^IP^(CF_3_)). This peak also contains a significant ν_a_(CF_2_) contribution. With an increase in the length of the perfluorinated moiety, the relative contribution of ν_a_(CF_2_) with respect to ν_a_^IP^(CF_3_) increases. Thus, for longer molecules (*N* > 6), the peak is more appropriately assigned to ν_a_(CF_2_) modes (see Figure 3A,B). The third strongest band appears at 1135 cm^–1^ and is assigned to the symmetric stretching vibration of the CF_2_ groups (ν_s_(CF_2_)). Most notably, the maximum peak heights of all three of these bands decrease with an increase in concentration (Figure 2A).

The calculated normal modes (Figure 2B) illustrate that ν(CF_*n*_) vibrations in PFAS are distinctly different from C–H stretching vibrations in hydrocarbons: Because fluorine atoms are heavier than carbon atoms, carbon atoms undergo displacements in ν(CF_*n*_) modes much larger than that of fluorine atoms. As a result, ν(CF_*n*_) modes couple with C–C stretching modes (ν(CC)) and with ν(CF_*n*_) vibrations of neighboring C–F groups such that the resulting normal modes are delocalized along the perfluorinated tail, indicating strong intramolecular vibrational coupling between C–F bonds. In contrast, C–H stretching vibrations in hydrocarbons are localized on single CH_*n*_ groups.

When quantifying concentration effects on the H2F4/TCE spectrum, special care must be taken to the proper normalization of the absorption spectra. As the concentration of H2F4 increases, the intensity of absorbance peaks also increases, following the Beer–Lambert law. At high concentrations, however, the absorption intensity does not increase linearly with concentration anymore.^47^ Furthermore, in ATR, penetration depth is affected by changes in the refractive index, which leads to a nonlinear dependency of the total intensity with sample concentration.^32,47^ To circumvent this problem when normalizing spectra to the H2F4 concentration, one can take the intensity of one of the peaks as a reference. Here, we use the integrated intensity of the ν_s_(CF_2_) peak for normalization. In Section S5 of the Supporting Information, we show spectra normalized to two other peaks: the rocking vibration ρ^IP^(CH_3_) around 1020 cm^–1^ and the ν_s_(CF_3_) band at 1353 cm^–1^. Normalization to all three bands leads to approximately the same concentration dependence in the C–F stretching spectral region, which suggests that the intensity of these modes remains largely unaffected by the environment, and thus, that these modes are suitable for normalization. DFT calculations in different environments, presented in Figure 5A, further support this claim. The advantage of using the ν_s_(CF_2_) band for normalization is that it is present in the spectra of all perfluoroalkyl substances and does not require the presence of other molecular groups.

After proper normalization, the observed decrease in peak height of ν(CF_*n*_) bands can be decomposed into two distinct effects, a broadening of the peak line widths and a decrease of the integrated intensity. To estimate both quantities, experimental spectra are fitted by a sum of Lorentzian distributions (see Section S4 of the Supporting Information). The integrated intensities of the ν_a_^OP^(CF_3_) and ν_a_^IP^(CF_3_) peaks normalized to the ν_s_(CF_2_) peak are shown in Figure 2C. In Figure 2D, we show line widths quantified by the full width at half-maximum of all three modes, ν_a_^OP^(CF_3_), ν_a_^IP^(CF_3_), and ν_s_(CF_2_). At low concentrations, integrated intensities and line widths of all modes remain unchanged, reflecting that all molecules are entirely solvated in the TCE and have identical environments. At higher concentrations, starting at around *x* = 0.01, we observe a decrease in integrated intensity of the ν_a_^OP^(CF_3_) and ν_a_^IP^(CF_3_) peaks. This is accompanied by an increase in line widths of all three peaks. The ν_a_^OP^(CF_3_) peak shows the strongest decrease in intensity and the highest increase in bandwidth at high concentration, which makes it the clearest marker of the fluorous phase. These results are robust with respect to the choice of fitting approach, as verified in Section S4 of the Supporting Information.

To assert whether the observed effect is specific to H2F4 or a general feature of perfluorinated molecules, we measured the spectrum of semi- (Figure 3A) and perfluorinated alkanes (Figure 3B) of different lengths in two limit cases, diluted in TCE and pure liquid. Molecules containing more than four fluorinated carbons are not fully miscible with TCE and undergo phase separation and therefore only low concentrations can be investigated. For all molecules, the integrated intensity of the ν_a_^OP^(CF_3_) peak is lower (Figure 3C, all red markers have lower values than corresponding blue markers) and its line width higher in the fluorous phase than in TCE solution (Figure 3D, red markers higher values than blue markers). Interestingly, the line width of the ν_a_^OP^(CF_3_) peak is independent of the chain length for both diluted per- and semifluorinated compounds (Figure 3D, blue dashed line), while we find linear trends with chain length in the case of pure liquids (Figure 3D, red dashed and dotted lines). In the Supporting Information Section S6, we show that also the bands corresponding to ν_a_^IP^(CF_3_) and ν_s_(CF_2_) undergo line broadening in pure liquids with respect to diluted TCE solution. Finding similar line broadening for all molecules under study suggests that there might be a single mechanism providing a unified explanation leading to line broadening for all of the ν(CF_*n*_) bands.

**Figure 4:**
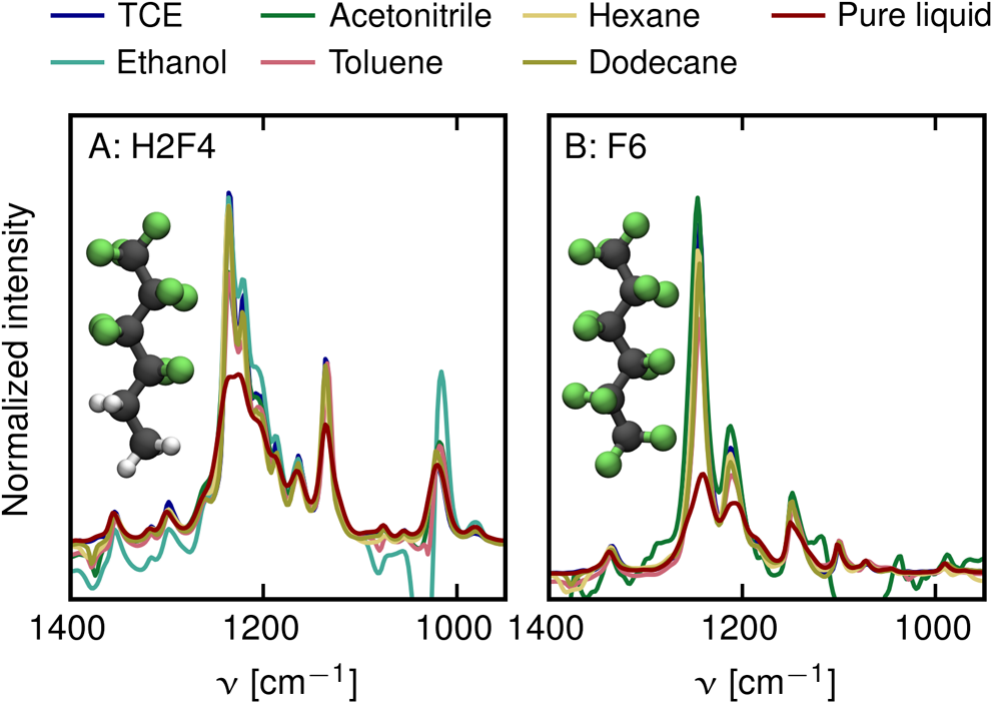
FTIR spectra of H2F4 (A) and F6 (B) at 10 mM concentration in different solvents. All spectra are normalized to the integrated intensity of the ν_s_(CF_2_) band.

To evaluate whether the observed spectral effects are specific to fluorous environments, spectra of dilute H2F4 in a set of different solvents are measured and compared to spectra of pure H2F4, see Figure 4A. The peak heights of ν_a_^OP^(CF_3_), ν_a_^IP^(CF_3_), and ν_s_(CF_2_) peaks at the maximum are only minimally affected by the choice of solvent. In particular, this is also true for the polar solvents ethanol (turquoise line) and acetonitrile (green line), despite the fact that the intensity of C–F stretching vibrations of isolated CF_*n*_ groups is known to be affected by the electrostatic properties of the solvation environment.^48^ The only solvent that leads to a significant decrease in peak heights is the aromatic solvent toluene (light red line), but the observed effect is small when compared to the effect in the fully fluorinated environment (dark red line). The same experimental approach was repeated for the fully fluorinated analog F6 (Figure 4B). In the same manner as for H2F4, also for F6 bandwidths and peak heights remain largely unaffected in all solvents. Only when molecules are exposed to a fluorous environment, the integrated intensities of ν_a_^OP^(CF_3_) and ν_a_^IP^(CF_3_) peaks decrease and an increase in bandwidths of ν_a_^OP^(CF_3_), ν_a_^IP^(CF_3_), and ν_s_(CF_2_) peaks is observed. These results have far-reaching implications for the interpretation of PFAS vibrational spectra. The minimal variation in C–F stretching peaks in solvents of different polarities, compared to the significant changes observed in the pure samples, suggests that the effect cannot be caused by the local electrostatic environment of individual molecules alone. Instead, the dynamic properties of molecules must be taken into account, a claim that will be further supported later in this work by theoretical calculations.

For molecules longer than 6 carbon atoms, the ν_s_(CF_2_) region shows multiple overlapping peaks, which may complicate the assignment of a unique integrated intensity for normalization as well as the determination of bandwidths. In such cases, the peak height ratio of ν_a_^OP^(CF_3_) and ν_s_(CF_2_) can be used as an alternative indicator of a fluorous environment (see Section S6 of the Supporting Information).

### Intensity Reduction in Fluorous Environment from Static DFT

**Figure 5:**
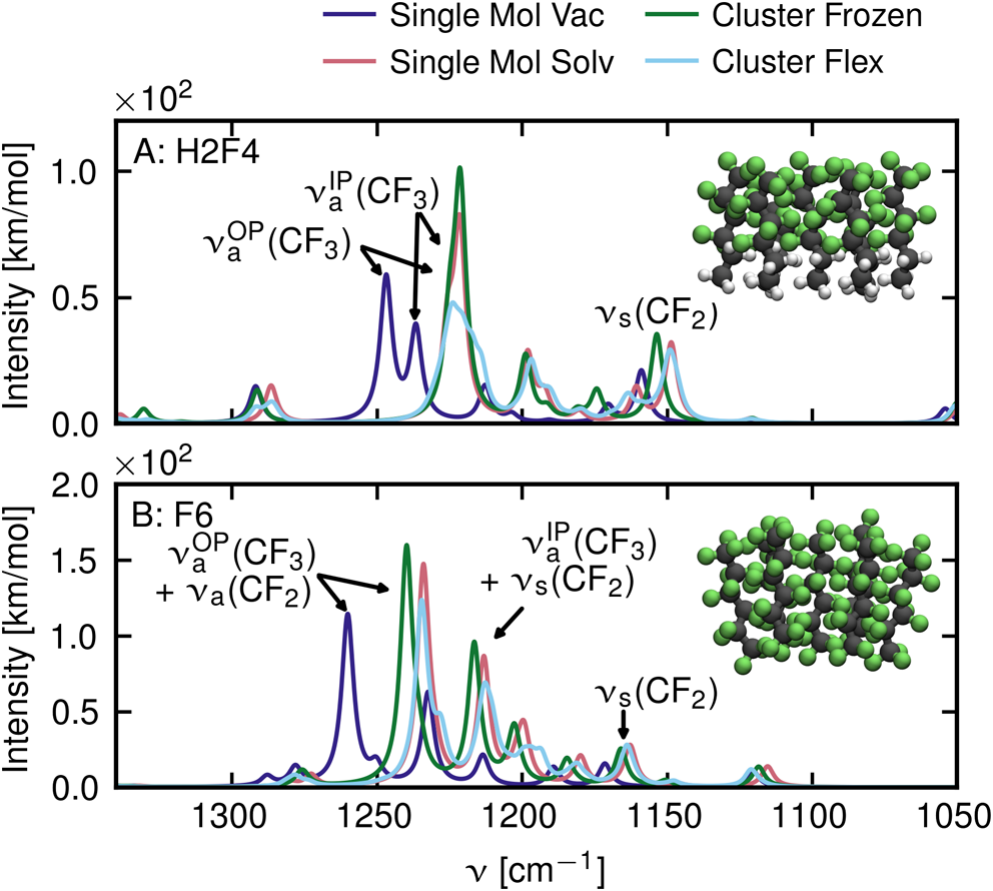
Vibrational spectra from static DFT calculations of H2F4 (A) and F6 (B) molecules. Vibration frequencies are determined for single molecules in vacuum (dark blue), single molecules in implicit solvent (red), hexagonal clusters of 7 molecules, where the outer shell of 6 molecules is kept frozen (green), as well as the same cluster where all molecules are allowed to move (light blue). All depicted spectra are broadened with Lorentzian line shapes with a full width at half-maximum of 5 cm^–1^.

In order to analyze the decrease of integrated intensity of CF_3_ stretching bands (cf. Figures 2C and 3C) we perform DFT-based geometry optimizations and subsequent vibrational analysis of the H2F4 and F6 compounds on the B97M-V/def2-TZVP^49,50^ level of theory. The B97M-V functional has recently been shown to reproduce interactions between perfluorinated molecules very reliably.^27^ We start out with the normal-mode analysis of single molecules in vacuum and add, piece by piece, more details of the molecular environment to reach an adequate level of description of the solvent environment. In total, we determine four different spectra for each compound: (1) a single molecule in vacuum. We then consider (2) a single molecule surrounded by an implicit solvent, parametrized to mimic bulk TCE, i.e., we embed the molecules in a homogeneously and isotropically polarizable background medium. To take into account inhomogeneities in the charge and polarizability distribution of the environment, we consider (3) a hexagonal cluster consisting of seven molecules of H2F4 or F6, respectively, again embedded in the same implicit solvent. In the normal-mode analysis, the outer shell consisting of 6 molecules is kept frozen and normal modes are determined only for the center molecule. In the final step (4), we account for intermolecular vibrational coupling by allowing molecules in the hexagonal shell to vibrate as well. The resulting spectra are shown in Figure 5 alongside optimized geometries of the considered clusters (depicted as insets). Note that for the resulting spectra, DFT eigenfrequencies are spread with Lorentzian functions with a constant width of 5 cm^–1^, since static DFT calculation does not give any insight into vibrational line shapes. Thus, integrated intensities and peak intensities are directly proportional to the shown spectra.

We observe a sizable red shift and an increase in the intensity of the ν_a_^OP^(CF_3_) and ν_a_^IP^(CF_3_) vibrations for both the H2F4 and F6 compounds, when adding an implicit solvent model (Figure 5, red lines) to the single molecule in vacuum (Figure 5, blue lines). Notably, in the case of H2F4, the bands are well separated in vacuum at frequencies of around 1246 and 1237 cm^–1^, while their frequencies nearly coincide around 1225 cm^–1^ when solvated. Other vibrations, like the ν_s_(CF_2_) or the low-frequency band around 1020 cm^–1^ corresponding to vibrations of the hydrogenated tail, (Figure 5A) also undergo red shifts but to a lesser degree. This indicates that the ν_a_^OP^(CF_3_) and ν_a_^IP^(CF_3_) vibrations are sensitive to changing the polarizability of the surrounding solvent from ε = 1, corresponding to vacuum, to ε = 2.5, corresponding to the dielectric constant of TCE.

The introduction of an inhomogeneous environment, i.e., placing the molecules into the interior of a frozen cluster of molecules (no displacement during normal-mode analysis) of the same type (green lines), however, leads to only minor further modifications of the spectra. For both the H2F4 and F6 compounds, the intensities of the ν_a_^OP^(CF_3_) and ν_a_^IP^(CF_3_) modes slightly increase with respect to solvated single molecules (red lines). For the F6 compound, we furthermore observe a slight blue shift of the same modes. The remainder of the displayed spectra is mostly unchanged. Only when we account for intermolecular vibrational coupling of the molecules in the cluster, by allowing for vibrational displacements in the entire cluster, do the resulting spectra (light blue lines) show pronounced decreases in intensity of the ν_a_^OP^(CF_3_) and ν_a_^IP^(CF_3_) modes compared to both solvated single molecules and single molecules embedded in frozen clusters. This effect is especially pronounced for the H2F4 compound (Figure 5A) but still sizable for the F6 compound in Figure 5B. The remaining IR active modes are largely unaffected by intermolecular coupling.

These calculations suggest that the decrease in intensity of the ν_a_^OP^(CF_3_) and ν_a_^IP^(CF_3_) modes, which we also find experimentally for perfluoroalkyl compounds in various solvents for increasing concentration (Figures 2 and 3), is caused by intermolecular vibrational coupling of linear perfluorinated molecules. In the Supporting Information Section S7, we provide further evidence for this claim. There, we show that intensity reductions of the ν_a_^OP^(CF_3_) and ν_a_^IP^(CF_3_) modes due to intermolecular vibrational coupling are robust against the choice of functional, for multiple popular DFT functionals. Furthermore, we show that the effect is specific to fluorous environments by comparing clusters with fluorinated with clusters with hydrogenated shells.

DFT results in Figure 5 predict negligible variations in the integrated intensity of the ν_s_(CF_2_) band in different environments. This suggests that the integrated intensity of this band is approximately proportional to the concentration of H2F4 or F6 in solution, supporting the argument we made earlier to use the integrated intensity of this band for normalization of absorption spectra.

### Line Broadening from FF-MD Simulations

While DFT-based normal-mode analysis can be used to calculate vibrational frequencies and IR absorption intensities, it does not yield information about the spectral line shapes. Optimally, to investigate these effects, one conducts DFT-based molecular dynamics simulations. In the present study, the long vibrational lifetimes of C–F vibrations require long simulation times (tens of nanoseconds), while the low F6 concentrations that are studied offer only little sampling, rendering DFT-based MD simulations prohibitively expensive. Instead, we rely on FF-MD simulations, employing the all-atom OPLS force field, that includes optimized parameters for both TCE and perfluorinated molecules with excellent bulk liquid properties.^13,40^ However, this choice comes with certain drawbacks. Nonpolarizable point-charge models are notoriously inaccurate at reproducing absorption intensities. Because of this, we focus on line shape effects only in the following analysis.

**Figure 6:**
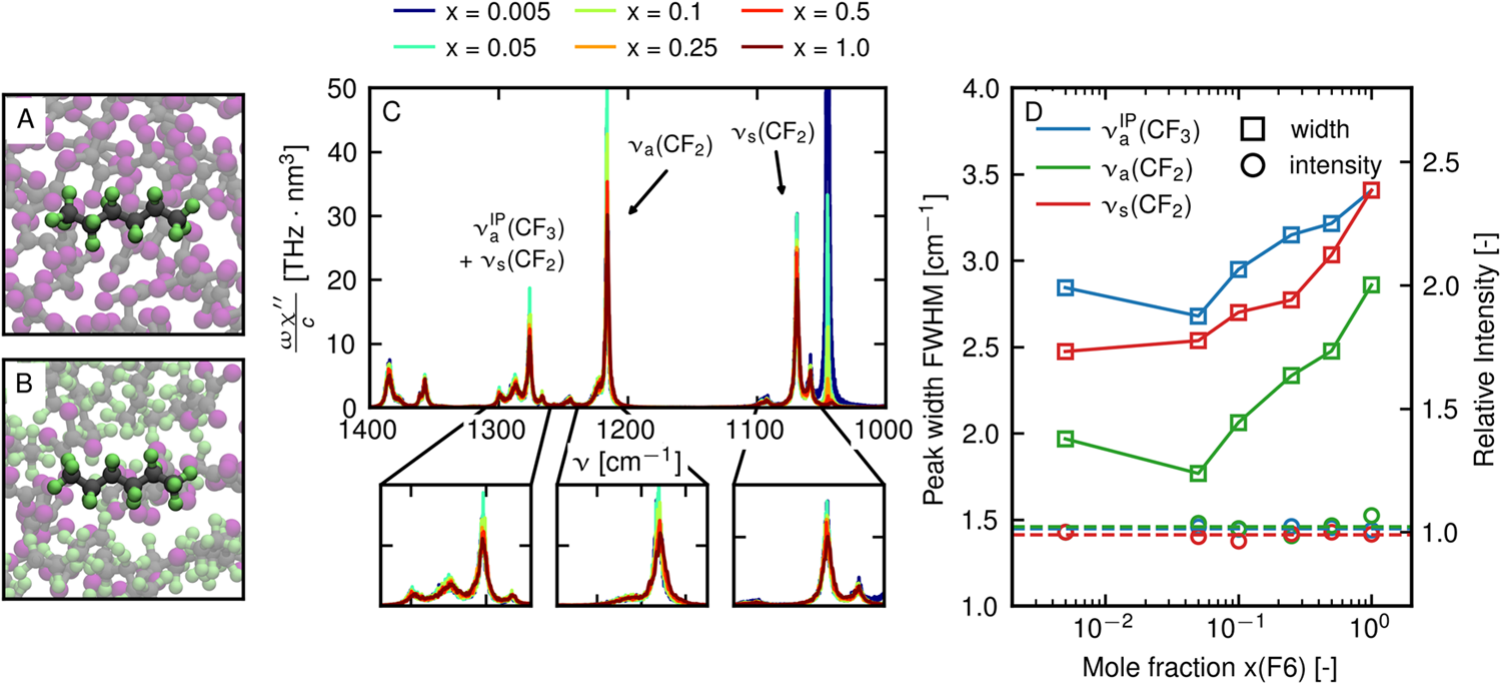
Absorption spectra of F6/TCE solutions at various concentrations from FF-MD simulations. Snapshots at mole fraction *x* = 0.005 and *x* = 0.5 are shown in (A, B), respectively. Absorption spectra normalized to the number of F6 molecules are shown in (C). As insets, we show close-ups of the spectral regions around the ν_a_^IP^(CF_3_), ν_a_(CF_2_), and ν_s_(CF_2_) modes. All modes are fitted by Lorentzian functions, and the resulting bandwidths (crosses), as quantified by the full width at half-maximum (FWHM), and integrated intensities (circles) are plotted as a function of concentration in (D). Dashed lines denote the mean relative intensities of different peaks.

**Figure 7:**
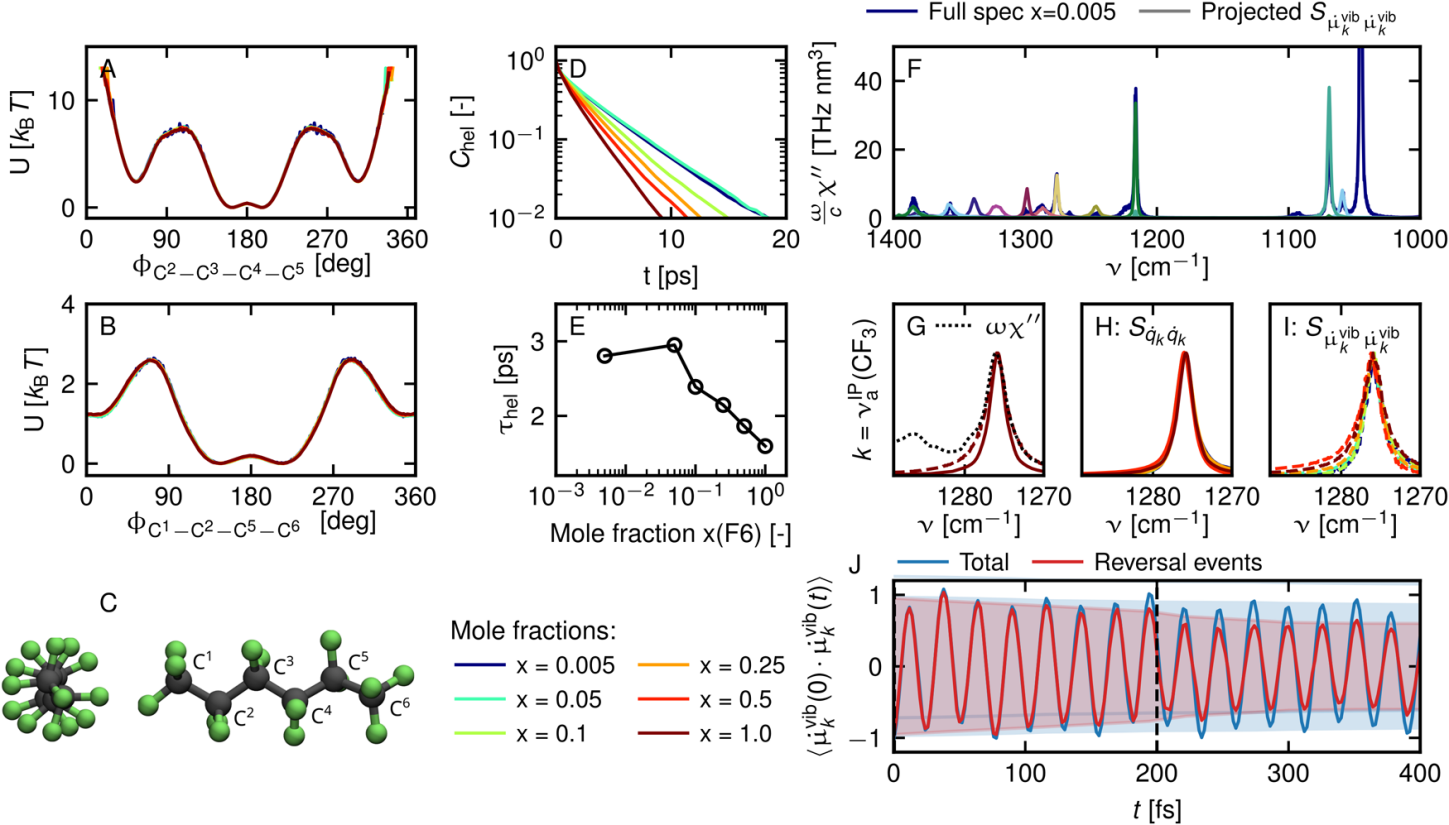
Molecular mechanism of line broadening from FF-MD simulations. (A, B) Free energy profiles of dihedral angles of the carbon backbone of F6 for different mole fractions of the F6/TCE mixtures. Carbon naming conventions are shown in the geometry-optimized structures shown in (C). (D) Autocorrelation functions of the helicity indicator in eq 7 for different mole fractions of F6/TCE mixtures, extracted autocorrelation times are shown in (E). (F) Absorption spectrum of *x* = 0.005 (dark blue line) decomposed into contributions from different vibrational modes (various other colors) based on projection in eqs 4 and 5. (G) Magnification of the spectral regime of the ν_a_^IP^(CF_3_) mode. The absorption spectrum (black dotted line) of pure F6 (*x* = 1.0) is compared to *S*_**μ̇**_*k*_^vib^**μ̇**_*k*_^vib^_ (dashed red line) and  of the same system both for the ν_a_^IP^(CF_3_) mode. (H) Concentration dependence of *S*_**μ̇**_*k*_^vib^**μ̇**_*k*_^vib^_. (I) Concentration dependence of . (J) Dipole autocorrelation function of the ν_a_^IP^(CF_3_) mode. We compare correlation functions that are averaged over the entire trajectory (blue line) with correlation functions averaged over subtrajectories where molecules undergo a reversal of backbone helicity at *t* = 200 fs.

We performed FF-MD simulations of F6 in TCE at varying concentrations. Representative snapshots of the highly diluted (*x* = 0.005) and of one highly concentrated (*x* = 0.5) system are shown in Figure 6A,B. From these simulations, we extract the susceptibility spectrum χ based on the autocorrelation function of the total dipole moment (see the Methods section for details). The energy absorption, which is proportional to ωχ″, where χ″ is the imaginary part of χ, is normalized to the perfluoroalkyl molarity *c*, see Figure 6C. In the displayed frequency regime, there are four peaks, which visually display significant dependence on the F6 concentration at frequencies of around 1045, 1069, 1218, and 1276 cm^–1^. The intensity of the lowest-frequency peak is proportional to the TCE concentration and therefore approximately inversely proportional to the F6 concentration and can thus be identified as a TCE mode. In the Supporting Information Section S8, we confirm this assignment by decomposing the overall absorption spectrum into contributions from different molecular types. The remaining three peaks stem from F6 and can be assigned with the help of normal-mode analysis (see Figure 7A and Supporting Information Section S1) as ν_s_(CF_2_), ν_a_(CF_2_) and the mixed ν_a_^IP^(CF_3_) + ν_s_(CF_2_) modes, from low to high frequency. Note that the intensities of FF-based normal modes are different from the normal modes according to static DFT calculations. In particular, the asymmetric CF_2_ stretching vibration occurs as one of the dominant modes, while in experiments and DFT-based spectra, this mode appears only weak and is thus not analyzed in Figures 2 and 3. However, experimentally, the ν_a_(CF_2_) mode does become a dominant mode for molecules with longer carbon chains such as F12 and it undergoes similar line broadening as the other C–F stretching modes (Figure 3B). Thus, the mode is experimentally relevant, and we will include it in the theoretical discussion from FF-MD simulations alongside the ν_a_^IP^(CF_3_) and ν_s_(CF_2_) modes. In the lower panels of Figure 6C, the spectral regions of the three modes are magnified. In agreement with the experimental results for H2F4 (Figure 2), the maximum peak heights of all three modes decrease visibly for increasing concentration of F6 in TCE. In order to extract line widths and integrated intensities, we fit the three peaks to Lorentzians. The results are shown in Figure 6D. The integrated intensities are approximately independent of F6 concentration. This is expected since for nonpolarizable force fields the transition dipole moment of a normal mode is independent of the electrostatic environment. The line width of the three discussed modes, however, increases substantially when going from diluted F6 in TCE to bulk liquid F6. This causes a decrease of the peak maximum intensity. The line width of a spectral feature is generally associated with the lifetime of the corresponding vibration. Increased line widths in an increasingly fluorinated environment therefore imply shorter vibrational lifetimes.

In order to investigate the molecular mechanism of the line broadening, we analyze the relevant vibrational modes in our FF-MD trajectories. Relating absorption spectra in the liquid phase to distinct molecular motion is challenging as collective molecular motion can lead to significant contributions to the spectrum. A notorious example for this is the highly collective dynamics of hydrogen bonds in liquid water.^51,52^ Thus, in a first step, we show in Supporting Information Section S8 that the line broadening observed in Figure 6C,D is indeed caused by a change in the vibrational dynamics of individual F6 molecules, as their environment changes, and not by collective vibrations of multiple molecules.

We then perform vibrational analysis of geometry-optimized single F6 molecules. The optimized structures (Figure 7C) show a helical twist of the carbon backbone, typical for perfluoroalkyl chains.^53^ This helical structure also persists in the liquid phase: Independent of concentration, the free energy profile of the middle dihedral angle of the carbon backbone of F6 in TCE mixtures (Figure 7A) displays four distinct minima corresponding to two distinct gauche (at 60 and 300°) and two distinct trans states (at 163 and 197°, corresponding to a helical twist of 17° per backbone dihedral). We characterize the overall helicity of an F6 molecule by the ϕ_C^1^–C^2^–C^5^–C^6^_ dihedral angle. The minima in the free energy profile (Figure 7B) reveal an overall helical twist of around 35°. Thus, two minimum energy configurations exist, corresponding to a left-handed and a right-handed helix, for which we perform normal-mode analysis separately. In order to analyze the vibrational dynamics from our F6/TCE mixture simulations related to individual normal modes, we project each molecule’s motion onto vibrational normal modes, taking into account the two different helicities; see the Methods section.

This results in the trajectories *q*_*k*_(*t*) of molecular motion for each normal mode from each individual molecule. The projection onto normal modes allows us to decompose the total dipole moment of a molecule **μ**(*t*) using
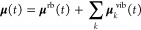
5into a rigid body rotation contribution **μ**^rb^(*t*) and vibrational contributions of each normal mode **μ**_*k*_^vib^(*t*), which are calculated from *q*_*k*_(*t*) and the transition dipole moments of the normal modes. Since rigid body rotations are slow compared to vibrations, they can be neglected in the IR range, and the energy absorption spectrum ωχ″(ω)∝ *S*_**μ̇μ̇**_(ω) can be decomposed into contributions from individual normal modes (Figure 7F) using
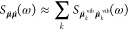
6where we use that different normal modes are approximately orthogonal to each other, see Supporting Information Section S10 for an exact derivation of the decomposition. In particular, the line shapes of the absorption spectrum are reproduced accurately in this decomposition (see Figure 7G, black dotted line ωχ″(ν) versus red dashed line *S*_**μ̇μ̇**_(ν) for the example of the ν_a_^IP^(CF_3_) mode).

When we assigned normal modes to the experimental spectra in Figure 2, we discussed that C–F_*n*_ stretching vibrations of perfluoroalkyl compounds are usually delocalized vibrations of the carbon backbone. Due to this, they are sensitive to the properties of the carbon backbone. To analyze this, we first decompose the overall absorption spectra into contributions from all-trans and gauche conformers in Supporting Information Section S9. This shows that gauche conformers do not contribute to the main ν(CF_*n*_) absorption peaks but, instead, introduce smaller additional peaks, which are slightly shifted in frequency with respect to the peaks corresponding to trans conformers. Thus, the line shape effects observed in Figure 7 are not caused by gauche conformers, and we disregard all gauche conformers in the calculation of the projected spectra shown in Figure 7F–I.

The helicity of the carbon backbone of perfluoroalkanes, however, is crucial for the understanding of their absorption spectra. This can be revealed by a careful analysis of the dipole moment decomposition in eqs 4–6. An exact decomposition of the vibrational power spectrum of molecular dipole moments *S*_**μ̇**^vib^**μ̇**^vib^_ (see Supporting Information Section S10 for details) reveals an interplay between molecular vibration and the change of helicity from left-handed to right-handed and vice versa, which we call helicity reversal. In particular, it contains terms due to molecular vibrations at fixed helicity, whose intensity is proportional to the absolute value of the transition dipole moment |**μ**_*k*_|, as well as terms related to the helicity reversal, which are proportional to the difference in the transition dipole moments |**μ**_*k*_^–^ – **μ**_*k*_^+^| between left- and right-handed helices. To identify the impact of helicity reversal on the line shape of the absorption spectrum we can set all terms containing information on helicity reversal to zero, by setting **μ**_*k*_^–^ – **μ**_*k*_^+^ = **0**, such that *S*_**μ̇**_*k*_^vib^**μ̇**_*k*_^vib^_(ω) reduces to |**μ**_*k*_^–^|^2^*S*_*q̇*_*k*_*q̇*_*k*__(ω). This corresponds to the spectral contribution of the *k*-th mode in the hypothetical case that upon helicity reversal, the geometry of the molecule does not change and the transition dipole moment of the normal mode is the same for both helicities.

In reality, however, the transition dipole moment rotates slightly upon helicity reversal. The impact of this rotation is given by the difference between *S*_**μ̇**_*k*_^vib^**μ̇**_*k*_^vib^_(ω) (Figure 7G,H dashed lines) and |**μ**_*k*_^–^|^2^*S*_*q̇*_*k*_*q̇*_*k*__(ω) (Figure 7G,H solid lines), which quantifies the spectral contribution of helicity reversals. Strikingly, *S*_*q̇*_*k*_*q̇*_*k*__(ω) has a narrower line width compared to ωχ″(ω) and *S*_**μ̇**_*k*_^vib^**μ̇**_*k*_^vib^_(ν) (Figure 7G) and *S*_*q̇*_*k*_*q̇*_*k*__(ω) does not reproduce the dependence of line width on F6 concentration in TCE (Figure 7H), while *S*_**μ̇**_*k*_^vib^**μ̇**_*k*_^vib^_(ω) does (Figure 7I). This suggests a speed-up of helicity reversal as the origin of the concentration-dependent line broadening of ν(CF_*n*_) modes of F6 in TCE observed in Figure 6.

The results of the spectral decomposition in Figure 7G–I shown for the example of the ν_a_^IP^(CF_3_) mode are qualitatively the same for the ν_a_(CF_2_) and the ν_s_(CF_2_) vibrations, see Supporting Information Section S13. In Figure 7J, we show direct proof that helicity reversal limits the lifetime of the ν_a_^IP^(CF_3_) vibration. We compare the autocorrelation function ⟨**μ**_*k*_^vib^(0)·**μ**_*k*_^vib^(*t*)⟩ for the *k* = ν_a_^IP^(CF_3_) vibration, when it is averaged over the entire trajectory (blue line), with the conditional autocorrelation function that takes only molecules into account, which undergo a helicity reversal at *t* = 200 fs (red line). The envelope functions of the resulting vibration, shown as shaded areas, show a loss of correlation during helicity reversal compared to the overall correlation function. This indicates that the vibrational dipole moment undergoes a rotation upon helicity reversal, which causes a dependence of the spectral line widths upon the characteristic time scale on which F6 molecules change their helicity.

To test the hypothesis that a speed-up of helicity reversal of F6 in fluorous environments causes line broadening directly, we introduce the autocorrelation function *C*_hel_(*t*) = ⟨θ_hel_(0)θ_hel_(*t*)⟩ of the helicity indicator function defined as
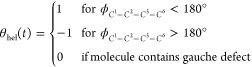
7Results for varying concentrations of F6 are shown in Figure 7D. Indeed, when extracting correlations times , we observe that the helicity lifetime τ_hel_ decreases from 2.9 ps for *x* = 0.005 to 1.5 ps for *x* = 1.0 (Figure 7E). It is important to note that this decrease in lifetime cannot be attributed to a reduced barrier height between the two helicities in the free energy profile of the relevant dihedral angle (Figure 7B). Indeed, not only the barrier height but also the entire free energy profile remains basically unchanged when changing F6 concentration. In Section S11 of the Supporting Information, we show, by comparing to transition state theory, that changes of the intramolecular energy landscape cannot account for the observed speed-up in helicity dynamics. Instead, it has to be caused by dissipative intermolecular coupling to the molecules environment.^54^

The speed-up of helicity reversal in the fluorous environment is not specific to F6 compounds. Perfluorinated carbon chains are known to exhibit a helical conformation for a range of different carbon-chain lengths.^53^ Indeed, DFT calculations presented in Section S12 indicate a helical conformation for perfluorocarbon chains as short as four perfluorinated carbon atoms. Only for even shorter carbon chains do the molecules exhibit a planar conformation. FF-MD simulations demonstrate a speed-up of helicity reversal also for the semifluorinated H2F4 compound when transitioning from diluted H2F4/TCE mixtures to the pure liquid (see Section S12). Consequently, line widths of C–F stretching vibrations, as calculated from FF-MD simulations, increase. These results suggest that the speed-up of helicity reversal of the carbon backbone is a universal effect for perfluoroalkyl chains in a fluorous environment, which provides a unified explanation of line broadening of C–F stretching vibrations in the fluorous phase.

## Conclusions

In this work, we provide evidence that the spectroscopic bands of the C–F stretching vibrations of perfluoroalkyl chains are highly sensitive markers of fluorous environments. Properly normalized, the peak heights of the respective bands are greatly reduced in fluorous environments compared to nonfluorous environments, which is related to substantial line broadening as well as an overall decrease in integrated absorption intensity. While the decrease in integrated intensity is observed only in some of the C–F stretching bands, line broadening is observed for all bands. This indicates a single mechanism behind the line broadening. Based on FF-MD simulations, we propose this mechanism to be a speed-up of conformational dynamics in fluorous environments: Perfluoroalkyl chains have a helical structure. Upon the reversal of helicity from left-handed to right-handed or vice versa, the transition dipole moment of C–F stretching vibrations rotates, limiting the vibrational lifetime. A speed-up of helicity reversal dynamics in fluorous compared to nonfluorous environments thus leads to the observed line broadening. The change in helicity is associated with a small change in the dipole moment of the PFAS molecules. Recently, it has been demonstrated that barrier crossing processes with exponential transition time distributions lead to distinct spectral signatures in absorption spectra,^55,56^ which, according to our predictions of helicity reversal time scales, would lie in the vicinity of 100 GHz. A thorough investigation of the coupling between PFAS helicity and CF stretch vibrations, for example, with the help of two-dimensional IR spectroscopy, could be a promising route for further research.

On a fundamental level, our work introduces C–F stretching vibrations as a unique class of IR vibrational probes, which defy the usual interpretation in the context of the electrostatic environment of C–F groups. Instead, their line shape is determined by the dynamic coupling between perfluoroalkyl chains. This can be inferred directly from experimental data based on the remarkable specificity of spectral changes to its environment: The impact of the electrostatic properties of the solvent on C–F stretching bands remains minimal compared to the spectral impact of fluorous environments. DFT normal-mode analysis and force-field molecular dynamics simulations support this interpretation.

On a practical level, we introduce C–F stretching vibrations and, in particular, the asymmetric out-of-plane CF_3_ stretching band ν_a_^OP^(CF_3_) around 1236 cm^–1^ as a vibrational marker of fluorous environments to be used as a tool for further studies of the fluorous phase. We expect that the reported spectroscopic markers will allow for the detection and characterization of nanosegregation and liquid–liquid phase separation of fluorinated fluids, as already small clusters of fluorinated molecules should show spectroscopic behavior distinct from the behavior of fully solvated molecules. To facilitate this, we report vibrational bandwidths of the ν_a_^OP^(CF_3_) band for molecules of different lengths, for both symmetrical F(*N*) molecules and H2F(*N*) molecules with alkylated tail groups and identify linear trends with the number of fluorinated carbons *N*, which can be used as reference for further studies.

## Data Availability

The data that support the findings of this study are available from the corresponding author upon reasonable request.

## Supporting Information Available

Raw experimental data, along with additional information on the postprocessing procedure: details on the employed Kramers–Kronig extraction of the transversal absorption spectrum, normal mode assignments, details on the fitting of spectral peaks and comparisons of spectra at different normalizations; comparison of DFT-based spectra with different exchange correlation functionals, and DFT-based spectra for clusters containing single H2F4 and F6 molecules in nonfluorinated solvents; FF-MD-based spectra of H2F4/TCE spectra, decompositions of FF-MD based computational spectra into contributions from fluorinated molecules and the TCE solvent, as well as the decompositions of spectra into contributions from trans and gauche conformers; and derivation of the spectral decomposition into contributions from helicity reversal and the resulting data for various vibrational peaks (PDF)
