# Supplementary material for: Infrared Spectroscopic Signatures of the Fluorous Effect Arise from a Change of Conformational Dynamics
Source: J Am Chem Soc. 2025 Mar 25;147(14):12040–50. doi: 10.1021/jacs.4c18434 (PMC11987022; doi:10.1021/jacs.4c18434)
Supplement: Supplementary file 1 — ja4c18434_si_001.pdf [file ja4c18434_si_001.pdf]

Supplementary material: Infrared Spectroscopic Signatures of the Fluorous Effect arise from a change of conformational dynamics

R. Cruz,[1,3] M. R. Becker,[2,3] J. Kozuch,[1] K. Ataka,[1] R. R. Netz,[2] and J. Heberle[1]

[1]*Experimental Molecular Biophysics, Freie Universität Berlin, Arnimallee 14, Berlin 14195, Germany*

[2]*Theoretical Bio- and Soft Matter Physics, Freie Universität Berlin, Arnimallee 14, Berlin 14195, Germany*

[3]*These authors contributed equally: R. Cruz, M. R. Becker.*

(Dated: February 13, 2025)

CONTENTS

S1. Normal mode assignment 2

S2. Kramers-Kronig correction of ATR spectra 3

S3. Raw ATR spectra of H2F4 in TCE solution 6

S4. Fitting of experimental data for parameter identification 7

S5. Normalization of experimental spectra 11

S6. Relative peak height dependency with molecular length 12

S7. Comparison of different XC functionals and hydrogenated clusters 13

S8. Decomposition of FF-MD spectra into single molecular and collective contributions 14

S9. FF-MD spectra of molecules with and without gauche deffects 15

S10. Expressing the energy absorption spectrum in terms of vibrational normal modes 15

S11. Dihedral helicity - Transition State Theory 18

S12. Helicity of short perfluoroalkyl chains 18

S13. Influence of helicity reversal on different spectral bands 19

References 19

S1. NORMAL MODE ASSIGNMENT

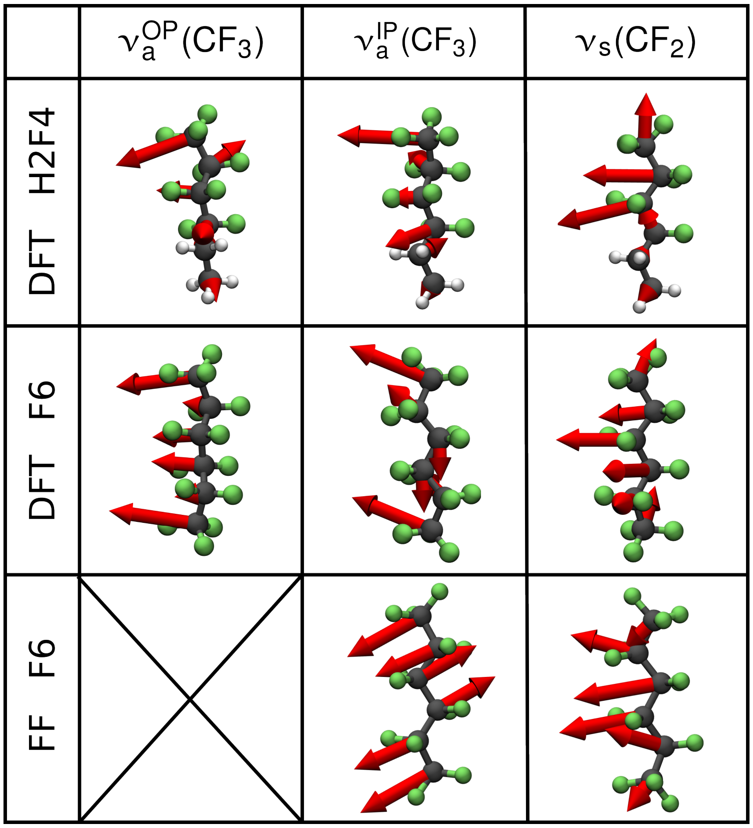

FIG. S1. Relevant normal modes of H2F4 and F6 molecules according to the B97M-V/def2-TZVP level of theory (DFT rows) and the OPLS-AA force-field (FF row).

S2. KRAMERS-KRONIG CORRECTION OF ATR SPECTRA

For convenience, Attenuated Total-Reflection (ATR) is used to measure samples at different concentrations, as this allows for fast exchange of liquid samples. The spectra obtained through this method, however, comprise a linear combination of transversal optic (TO) and longitudinal optic (LO) energy loss functions.[1] The same functions dominate the shape of normal-incidence spectra measured in transmission and in reflection, respectively. Hence, ATR spectra can be interpreted as a combination of a transmission-like (TO) spectrum and a reflection-like (LO) spectrum.

At high sample concentrations, the increase in the relative weight of the reflection-like component leads to a red-shift of IR peaks and to an increase in the relative intensity of low-frequency bands S2. This effect is characteristic of molecules with high absorptivity, such as perfluoroalkanes.[2]

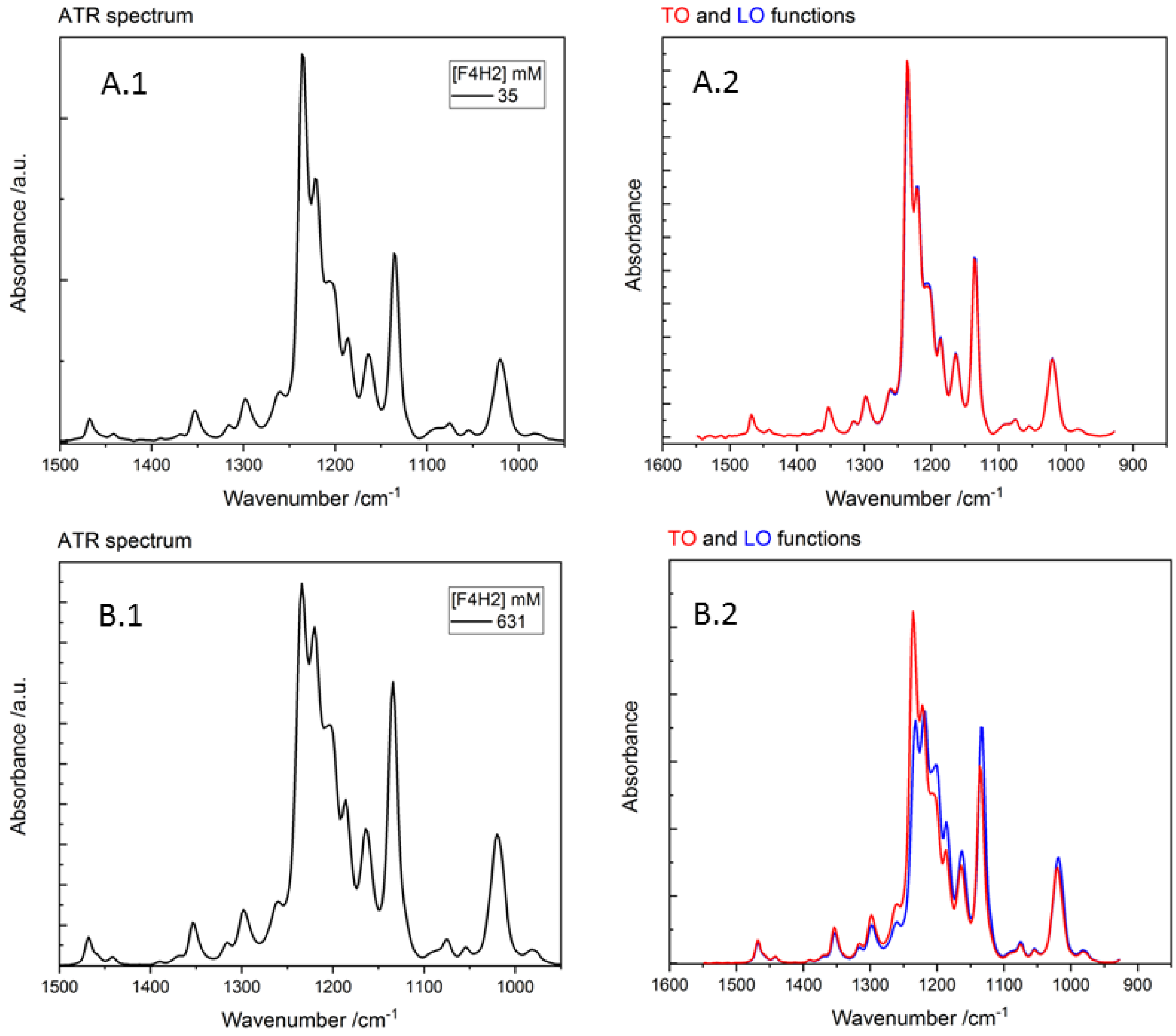

FIG. S2. Raw ATR spectra of H2F4 solution in TCE at 35 mM (A.1) and 631 mM (B.1). At increasing H2F4 concentration and higher absorbance values, spectra get distorted because of the underlying TO-LO split. The transmission-like component can be extracted by using Kramers-Kronig relations (A.2) and (B.2).

The spectral components TO and LO can be extracted from the reflectance spectrum using Kramers-Kronig relations. The method was first developed by Plaskett and Schatz[3] for measurements in reflection through a transparent window and later applied to ATR measurements by Bardwell and Dignam.[4] In a typical ATR experiment, the vibrational response of a sample is

recorded as an absorbance spectrum (A). It can be converted to a reflectance spectrum R by using the expression

$$R = e^{-A\log 10}, \tag{S1}$$

where log is the natural logarithm. The complex reflection coefficient ($\boldsymbol{r}$) of an interface is related to its reflectance R by a frequency-dependent phase ϕ according to

$$\boldsymbol{r}(\nu) = \sqrt{R(\nu)}e^{i\phi} \tag{S2}$$

with frequency ν. In this section only, we use bold characters to represent complex quantities (in later sections they will be used to represent vectors instead). The reflectance $R(\nu)$ can be related to $\phi(\nu)$ via a Kramers-Kronig relation by introducing a complex frequency $\boldsymbol{\omega}$ and expressing the logarithm of $\boldsymbol{r}(\boldsymbol{\omega})$

$$\log \boldsymbol{r}(\boldsymbol{\omega}) = \frac{1}{2}\log R(\boldsymbol{\omega}) + i\phi(\boldsymbol{\omega}), \tag{S3}$$

with $R(\boldsymbol{\omega}) = |\boldsymbol{r}(\boldsymbol{\omega})|^2$. Assuming that $\boldsymbol{r}(\boldsymbol{\omega})$ is analytic in the upper half-plane, the integral of $\log \boldsymbol{r}(\boldsymbol{\omega})$ along the contour enclosing the upper half-plane should be zero:

$$\int_\Gamma \frac{\log \boldsymbol{r}(\boldsymbol{\omega})}{\boldsymbol{\omega}'^2 - \boldsymbol{\omega}^2} d\boldsymbol{\omega}' = 0. \tag{S4}$$

This integral can then be expressed in terms of R and ϕ using Eq. S3 and resolved using Cauchy's residue method. Depending on the incidence angle and the refractive indices of the window and sample, the function $\frac{\log \boldsymbol{r}(\omega)}{\omega'^2 - \omega^2}$ may exhibit poles on the imaginary axis, which must be considered, when choosing the integration contour. The generalized relation

$$\phi(\nu) = \phi_0 - P\frac{\nu}{\pi}\int_0^\infty \frac{\log R(\nu')}{\nu'^2 - \nu^2} d\nu', \tag{S5}$$

between ϕ and R can be obtained by incorporating a phase offset ϕ_0 dependent on the optical configuration.[3,4] Plaskett and Schatz distinguished 3 cases to evaluate the phase offset depending on whether (1) $\sin^2(\alpha) < \frac{1}{\varepsilon_{Si}}$, (2) $\frac{1}{\varepsilon_{Si}} < \sin^2(\alpha) < \frac{\varepsilon_{H_yF_x}}{\varepsilon_{Si}}$ and (3) $\sin^2(\alpha) > \frac{\varepsilon_{H_yF_x}}{\varepsilon_{Si}}$, the last corresponding the the case of total reflection. Taking the literature values for silicon (11.7), perfluorohexane (1.57) and an incidence angle of 27°, $\sin^2(\alpha) \approx 0.20$ lies close to the critical value $\frac{\varepsilon_{H_yF_x}}{\varepsilon_{Si}} \approx 0.13$, and therefore between cases (2) and (3). Because of this, we opted to estimate the offset value from the direct comparison of spectra measured in transmission with spectra calculated from ATR. A phase offset of $\frac{\pi}{2}$ was found to give accurate results and therefore used for all calculations. Taking

$$\phi(\nu) \approx \frac{\pi}{2} - \frac{\nu}{\pi}\int_0^\infty \frac{\log R(\nu')}{\nu'^2 - \nu^2} d\nu'. \tag{S6}$$

the transmission-like spectrum TO is calculated from R and ϕ in four steps. First, $\log R(\nu')$ is obtained from the absorbance spectrum recorded in ATR configuration (A) as $\log R(\nu') = -A \cdot \log 10$ and ϕ is calculated using Eq. S6. Second, we calculate the complex refractive index of the sample $\boldsymbol{n}$ from R and ϕ using Fresnel relations. Third, the complex dielectric constant of the sample $\boldsymbol{\varepsilon}(\nu)$ is calculated from the refractive index $\boldsymbol{n}$. Lastly, we retrieve TO and LO from $\boldsymbol{\varepsilon}(\nu)$ using their definitions S7.

$$\log R(\nu'),\, \phi(\nu) \quad \rightarrow \quad \boldsymbol{n} = n + ik \quad \rightarrow \quad \boldsymbol{\varepsilon}(\nu) \quad \rightarrow \quad TO = Im(\boldsymbol{\varepsilon}(\nu)),\, LO = Im\left(\frac{-1}{\boldsymbol{\varepsilon}(\nu)}\right) \tag{S7}$$

To calculate $\boldsymbol{n}$ from $R(\nu')$ and $\phi(\nu)$, we used the normal-incidence version of Fresnel equations S8. Under this approximation, we do not need to distinguish between s and p polarization, such that

$$\boldsymbol{r}(\nu) = \frac{(\boldsymbol{n} - n_{Si})}{(\boldsymbol{n} + n_{Si})} = \sqrt{R(\nu)}e^{i\phi}. \tag{S8}$$

Assuming a non-absorbing window, the refractive index n_{Si} is real in the whole frequency domain. Equation S8 can be solved to express $\boldsymbol{n} = n + ik$ as a function of the reflectance R and the phase ϕ. By taking the real and imaginary parts, we obtain an expression for n and k (S9, S10).

$$n(\nu) = \frac{1 - R(\nu)}{1 - 2\sqrt{R(\nu)}\cos\phi(\nu) + R(\nu)} \tag{S9}$$

$$k(\nu) = \frac{2\sqrt{R(\nu)}\sin\phi(\nu)}{1 - 2\sqrt{R(\nu)}\cos\phi(\nu) + R(\nu)} \tag{S10}$$

The dielectric constant of the sample is then obtained from the complex refractive index using relation S11

$$\boldsymbol{\varepsilon}(\nu) = \boldsymbol{n}^2(\nu) = (n(\nu) + k(\nu)i)^2 \tag{S11}$$

Finally, the transmission (TO) and reflection-like (LO) spectra are obtained from the complex dielectric constant from their definitions S12.

$$TO = Im(\boldsymbol{\varepsilon}(\nu)) \qquad LO = Im\left(\frac{-1}{\boldsymbol{\varepsilon}(\nu)}\right) \tag{S12}$$

To demonstrate the validity of the approach, the TO spectrum, calculated from the ATR spectrum of a pure H2F10 sample using Kramers-Kronig relations, is compared with a transmission spectrum of H2F10 crystals dispersed in a KBr pellet (Fig. S3, A). The red-shift and the relative higher intensity of the low-frequency bands observed in ATR were almost entirely caused by the reflection-like component and not by the molecular environment, as one could initially infer.

In Fig. S3 B, the TO spectrum of pure decane, also obtained through Kramers-Kronig analysis of raw ATR data, is compared with a spectrum recorded at low concentrations in TCE (S3, A). Their similarity indicates that decane molecules are exposed to a very similar chemical environment in both, TCE-solution and pure form.

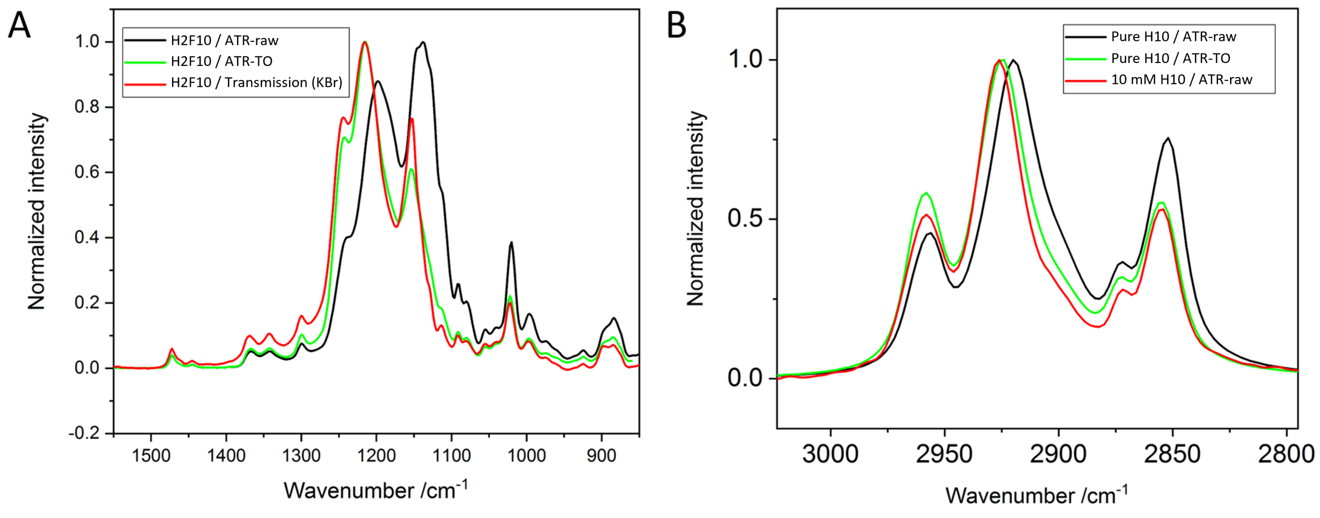

FIG. S3. (A) FT-IR spectra of ethyl-perfluorodecane (H2F10). Raw ATR spectra shows strong distortion. TO calculated function reproduces the shape of the same compound measured in transmission in a KBr pellet. (B) FT-IR spectra of decane. Raw ATR spectrum of pure decane shows some distortion before correction (black). TO function after Kramers-Kronig analysis (green) reproduces the shape of the ATR spectrum recorded at low concentration in TCE solution (red).

S3. RAW ATR SPECTRA OF H2F4 IN TCE SOLUTION

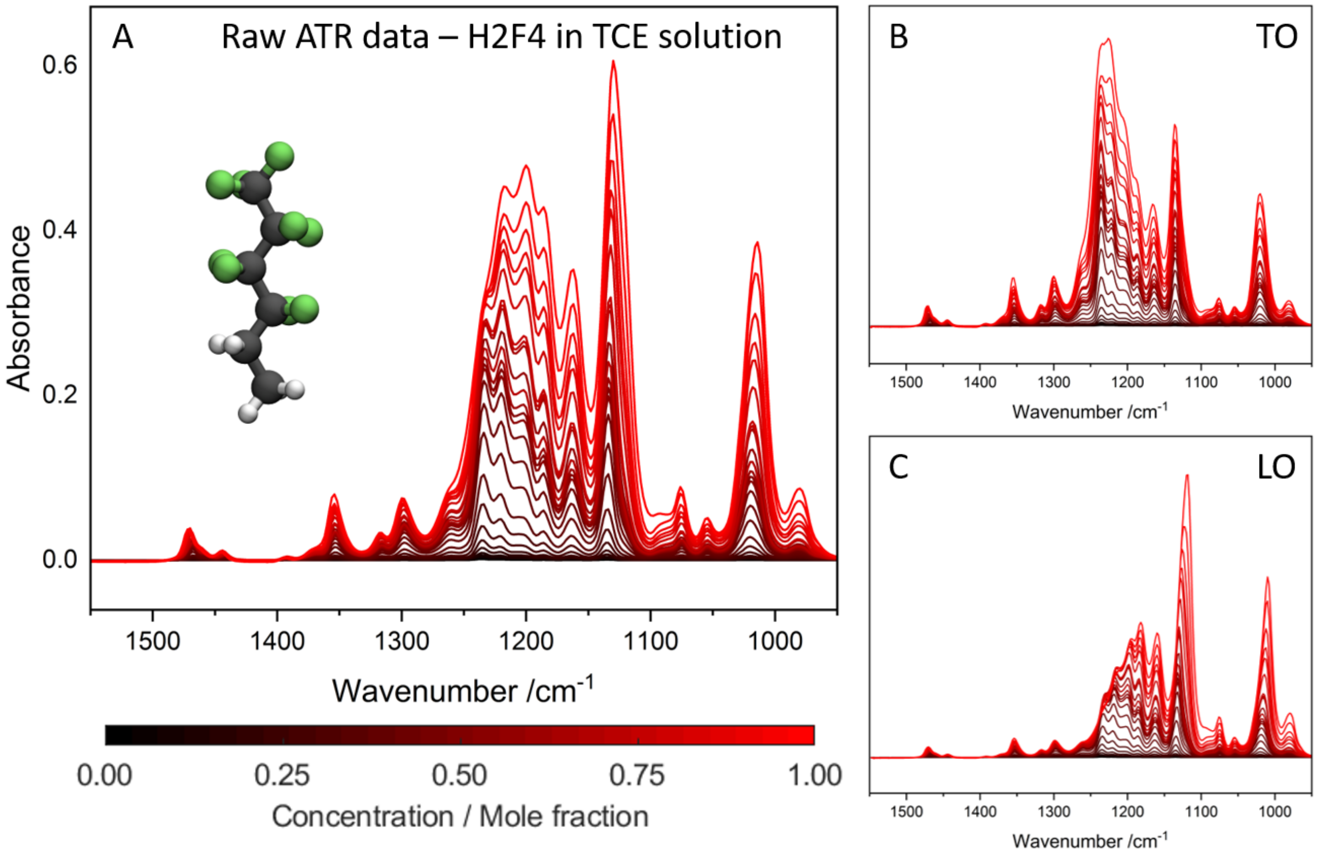

FIG. S4. (A) Raw ATR spectra of H2F4 in TCE solution at different concentrations and TO (B) and LO (C) functions after Kramers-Kronig analysis (non-normalized).

S4. FITTING OF EXPERIMENTAL DATA FOR PARAMETER IDENTIFICATION

Peak-fitting was performed in a two-steps process. First, the frequencies corresponding to peak maxima are identified using a continuous wavelet transform. In this method, the half-width at half maximum (HWHM) of the wavelet is a free parameter, which should be selected to approximately match that of the peaks in the original spectrum. As wavelet, we use the second derivative of a Lorentzian distribution S13.

$$\psi(\omega',\omega) = \frac{-1}{\pi\gamma}\frac{d^2}{(d\omega')^2}\left(\frac{1}{1+(\frac{\omega'-\omega}{\gamma})^2}\right). \tag{S13}$$

Where ω' represents the wavenumber domain, ω the wavenumber center and γ the HWHM of the Lorentzian distribution. The transform is the convolution of the wavelet and the original spectrum ($y(\omega')$), obtained by translating the wavelet over the entire wavenumber domain of the original spectrum S14 (see Fig. S5).

$$\tilde{y}(\omega) = \int_{\omega_1}^{\omega_2} y(\omega')\psi(\omega',\omega)\,d\omega' \tag{S14}$$

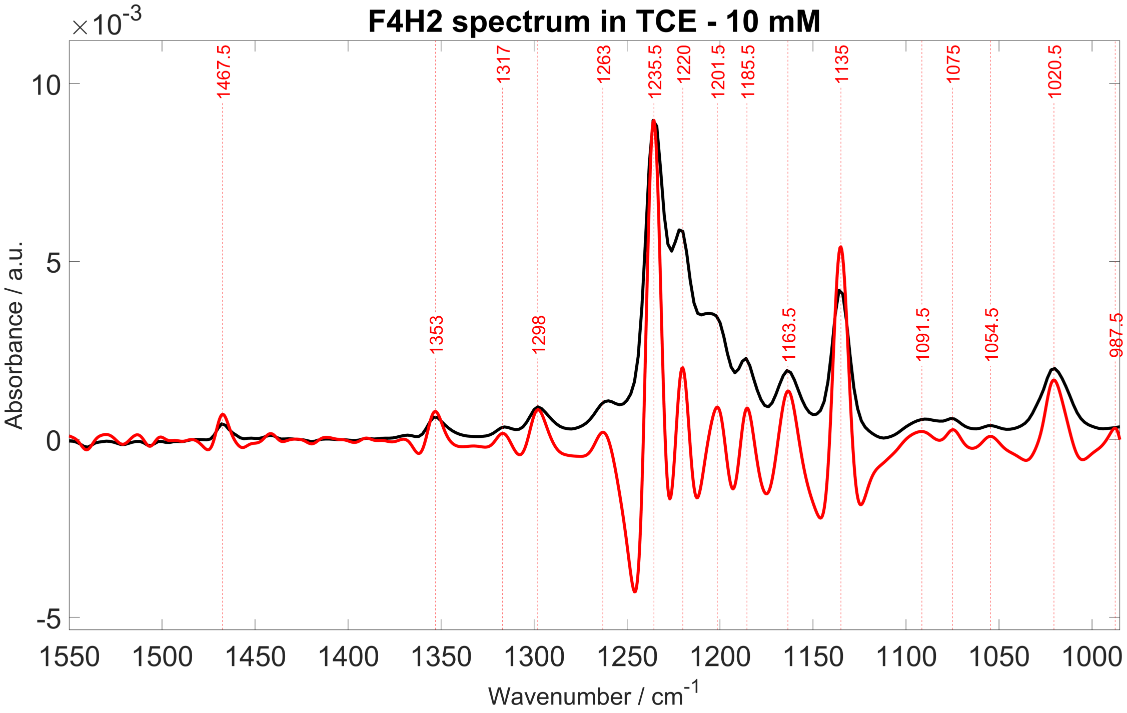

FIG. S5. FT-IR spectrum of H2F4/TCE solution at c = 10 mM ()black and its continuous wavelet transform (red) using $\gamma = 5$ cm^{-1}.

Peak positions are identified by scanning for local maxima in the wavelet transform. To filter out noise contributions, local extrema are assigned to a peak only if their intensity exceeds 2% of the absolute maximum intensity. This procedure ensures that the number of peaks assigned to the spectrum is determined systematically. The only choice of parameters is the intensity threshold and the wavelet's HWHM. We used a value of 5 cm^{-1} for the HWHM.

The original spectrum is then fitted using a linear combination of Lorentzian distributions centered at the so-obtained peak maxima (ω_n) S15. Peak positions are kept constant and integrated intensities (A_n) and HWHMs (γ_n) are found by fitting.

$$y_{fit}(\omega) = \sum_{n=1}^{N}\frac{A_n}{\pi\gamma_n}\left(\frac{1}{1+(\frac{\omega-\omega_n}{\gamma_n})^2}\right). \tag{S15}$$

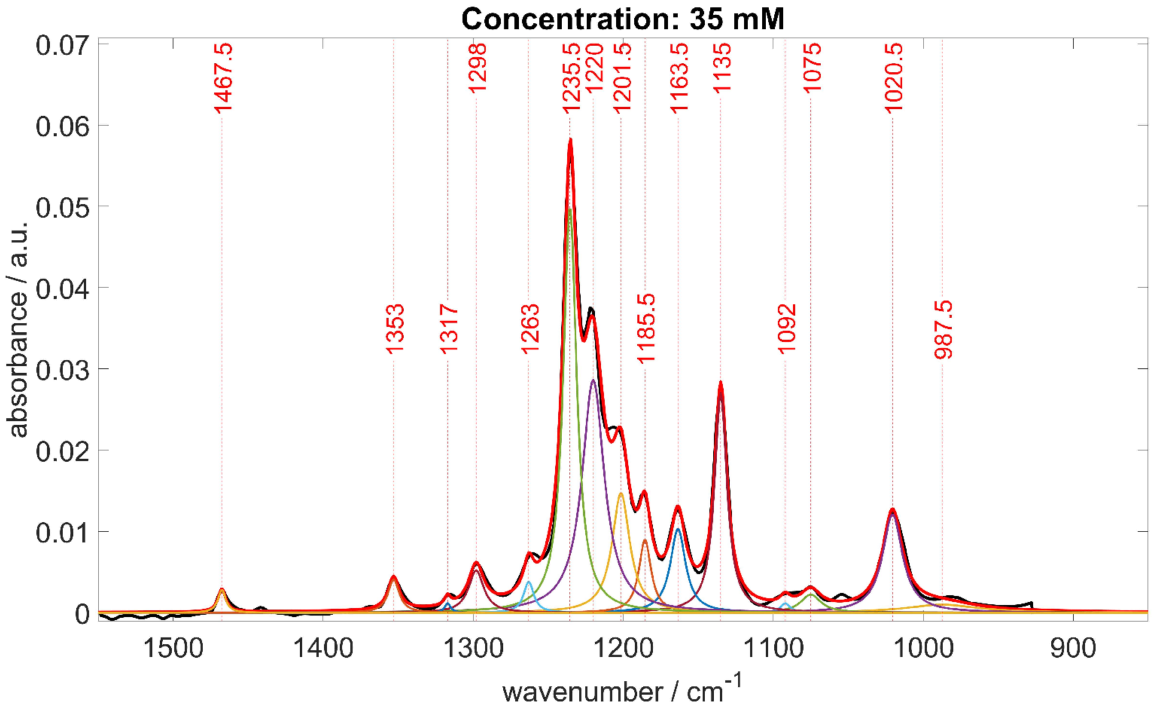

FIG. S6. Example fit of H2F4/TCE solution at $c = 35$ mM with a sum of Lorentzian functions. Raw data is shown in black, the total fit in red, and individual contributions to the fit in various colours.

To verify that the decomposition of spectra in terms of overlapping Lorentzians is unique, we also implemented a second fitting procedure. In this approach, we do not constrain the peak positions, but instead seek to determine a global optimum of the mean square deviation using a stochastic simulated annealing algorithm. With this procedure, fitting also the smaller peaks from the spectrum might yield varying results, such that we chose a smaller number of total Lorentzian functions. The results of the constrained and unconstrained fits are compared in Figs. S7. In the spectral region where peaks are strongly overlapping - between 1100 and 1250 wavenumbers - unconstrained fitting results in a decomposition of the absorption spectrum into Lorentzians with nearly the same center frequencies as those selected according to the wavelet transform discussed earlier. This indicates that the decomposition into Lorentzian functions leads to a unique and globally optimal fit of the absorption spectrum. In Fig. S8 we compare the line widths and peak intensities reported in Fig. 2 of the main text, this time determined from the two different fitting procedures. Both fitting methods demonstrate the same trends: the line width of $\nu_a^{OP}(CF_3)$, $\nu_a^{IP}(CF_3)$ and $\nu_s(CF_2)$ bands increases with increasing H2F4 concentration, while the relative intensities of the $\nu_a^{OP}(CF_3)$ an ν^{IP} bands decrease. This underscores that the effects we describe in the main text are robust against changes in the fitting procedure. The only observable difference between the two fitting schemes is an outlier in the $\nu_s(CF_2)$ band around x(H2F4)=0.2, which can be expected from an automated stochastic fitting, as well as a quantitative difference in the $\nu_a^{IP}(CF_3)$ band: the unconstrained fitting results in overall smaller intensities in this band compared to constrained fitting, which, however, follows the same trend with H2F4 concentration.

In order to asses the influence of the peak shapes employed for fitting, constrained fitting was repeated using a sum of Gaussian distributions instead of Lorentzians. Peak positions were found using the second derivative of a Gaussian distribution with $\sigma = 2.5$ cm^{-1} as wavelet and an intensity threshold of 2% as before. Integrated intensities and FWHMs were found, by fitting a linear combination of Gaussians centered at those maxima. Results are shown in Fig. S8. Using Gaussians instead of Lorentzians leads to overall slightly worse fits, in particular in the overlapping region. Nevertheless, the trends of decreasing intensity and increasing linewidths with increasing H2F4 concentration remain robust with somewhat smaller concentration effects.

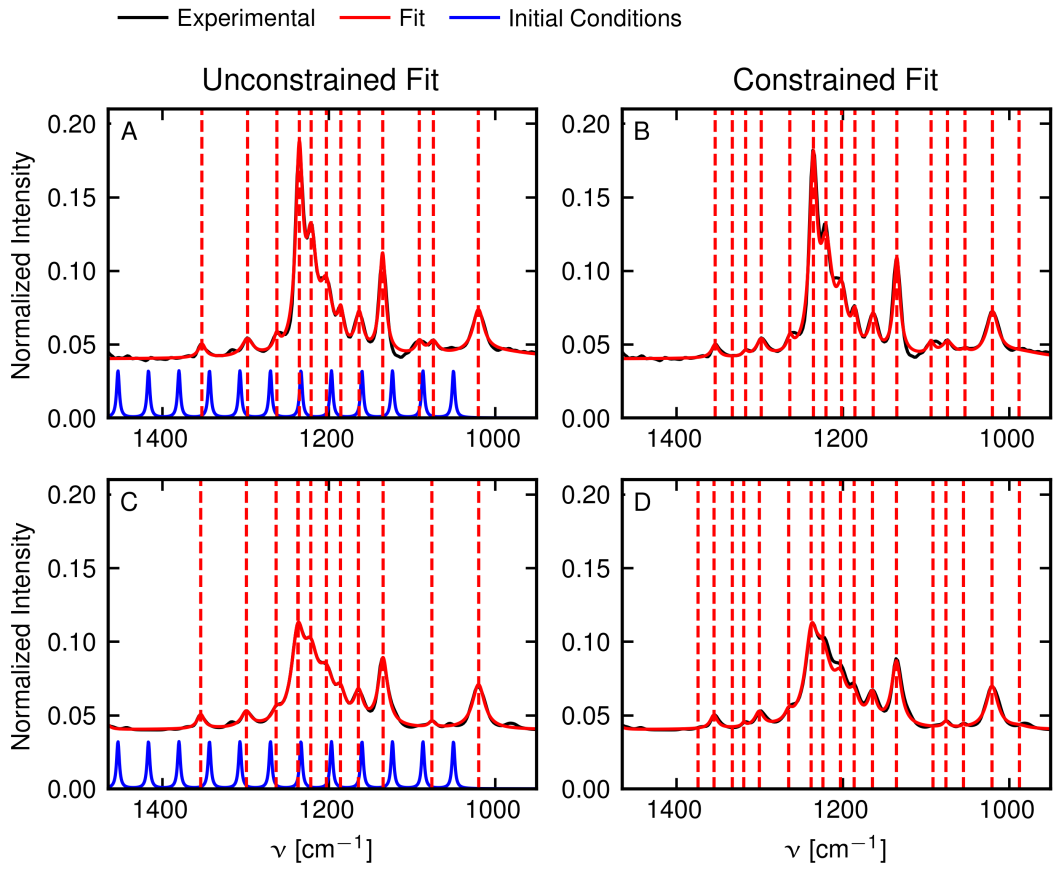

FIG. S7. Comparison of spectral fitting of H2F4/TCE mixtures at dilute (x(H2F4)=0.0002, panels **A** and **B**) and high concentrations (x(H2F4)=0.24 panels **C** and **D**). Peak positions are indicated by vertical dashed lines.

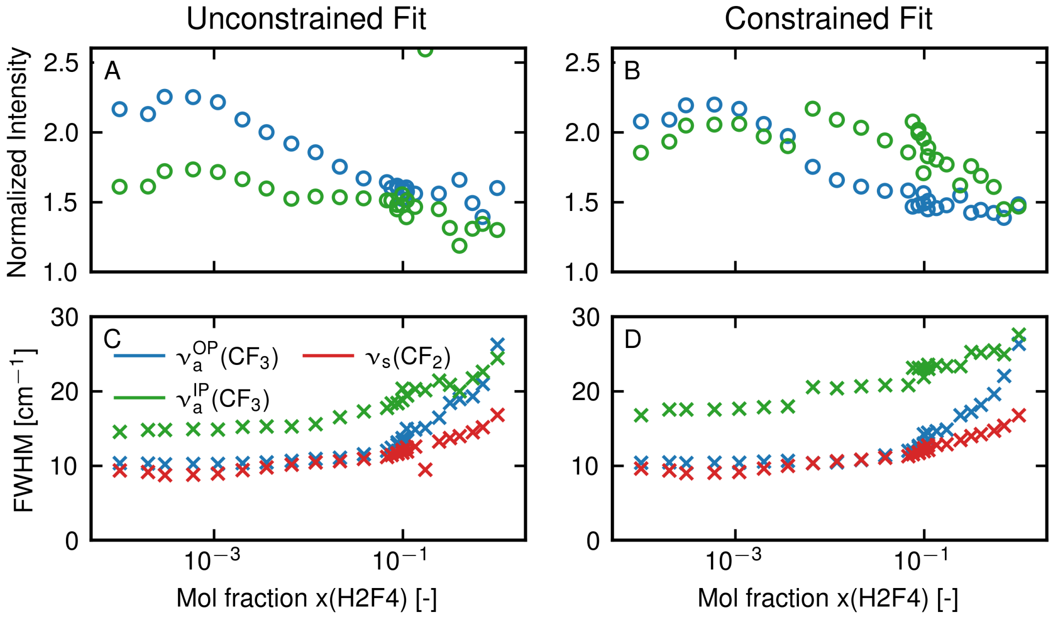

FIG. S8. Comparison of intensities and line widths determined by fitting procedures with constrained and unconstrained peak positions as a function of H2F4 concentration.

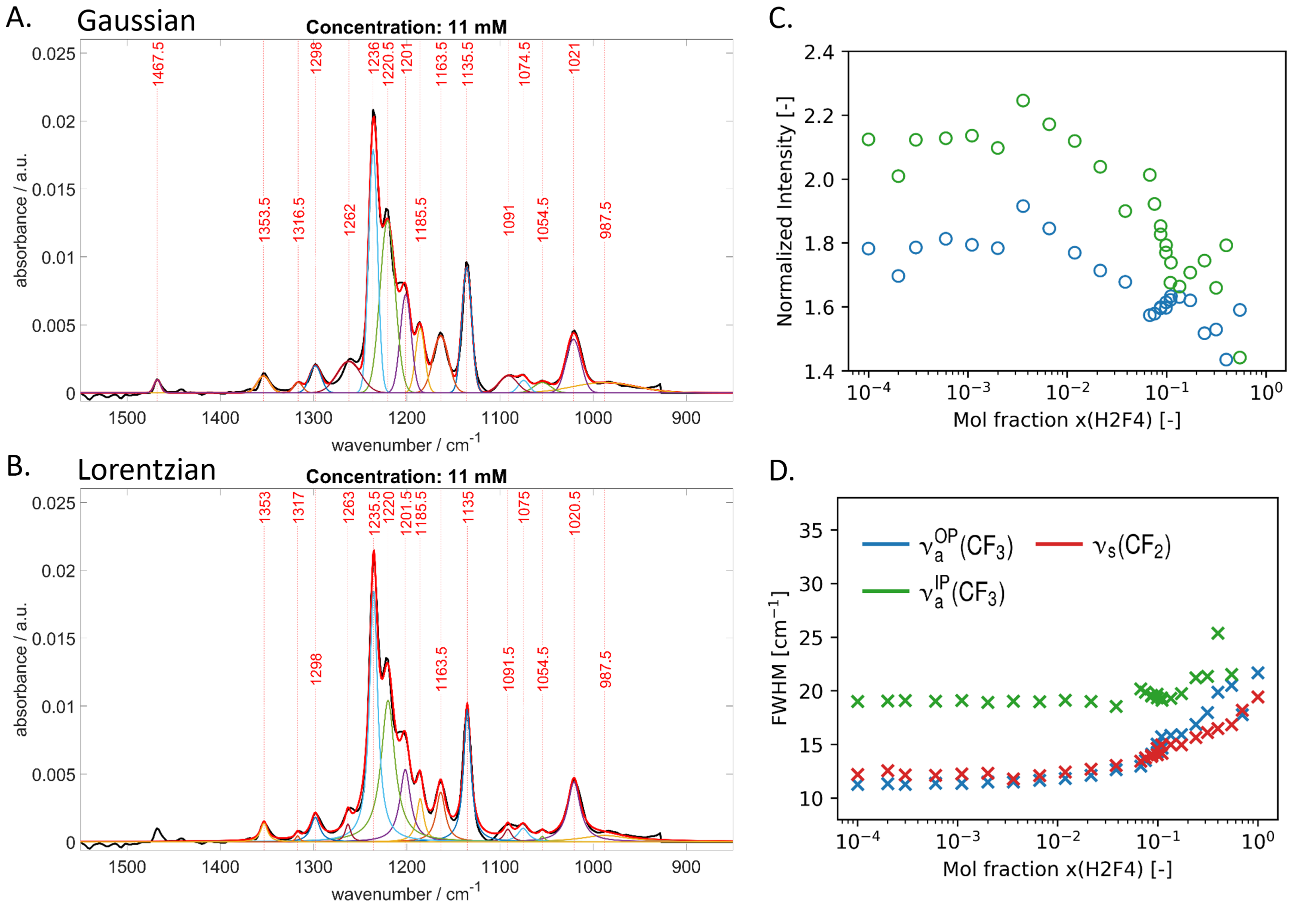

FIG. S9. Comparison of a constrained fit using a set of Gaussian distributions (A) versus Lorentzian distributions (B) for H2F4 in TCE at 11 mM concentration. Relative intensities (C) and line widths (D) at different molar ratios from the constrained fit using Gaussian functions.

S5. NORMALIZATION OF EXPERIMENTAL SPECTRA

To investigate the validity of the normalization of our spectra with respect to the $\nu_s(CF_2)$ band chosen in the main text, we compare the resulting spectra spectra of H2F4/TCE mixtures (Fig. S10 A) with spectra normalized to the integrated intensity of two other peaks (Fig. S10 B,C). In particular we compare to the normalization with respect to the $\rho^{IP}(CH_3)$ rocking mode (Fig. S10 B), which, since it does not involves the fluorinated part of the backbone, we assume to be independent of the fluor-content of the molecular environment. All three displayed spectra show the same trends with H2F4 concentration, suggesting that the bands used for normalization follow a universal scaling with H2F4 concentration and thus are suitable for normalization.

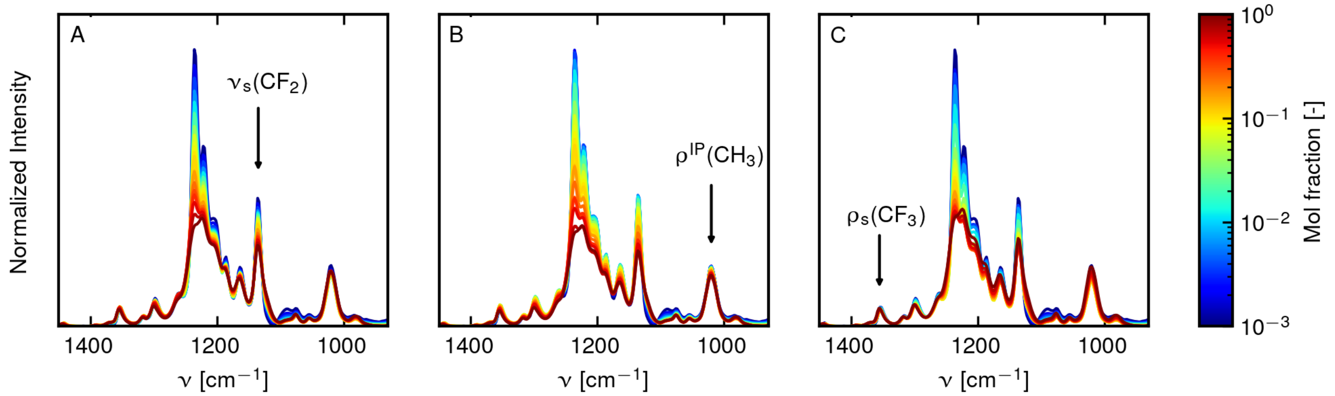

FIG. S10. FITR spectra of H2F4/TCE solutions at various concentrations of H2F4 normalized towards the intensity of the $\nu_s(CF_2)$ mode (**A**), the twisting mode of the alkylated headgroup $\rho^{IP}(CH_3)$ and the $\nu_s(CF_3)$ mode. Peaks that were used for normalization are indicated by arrows.

S6. RELATIVE PEAK HEIGHT DEPENDENCY WITH MOLECULAR LENGTH

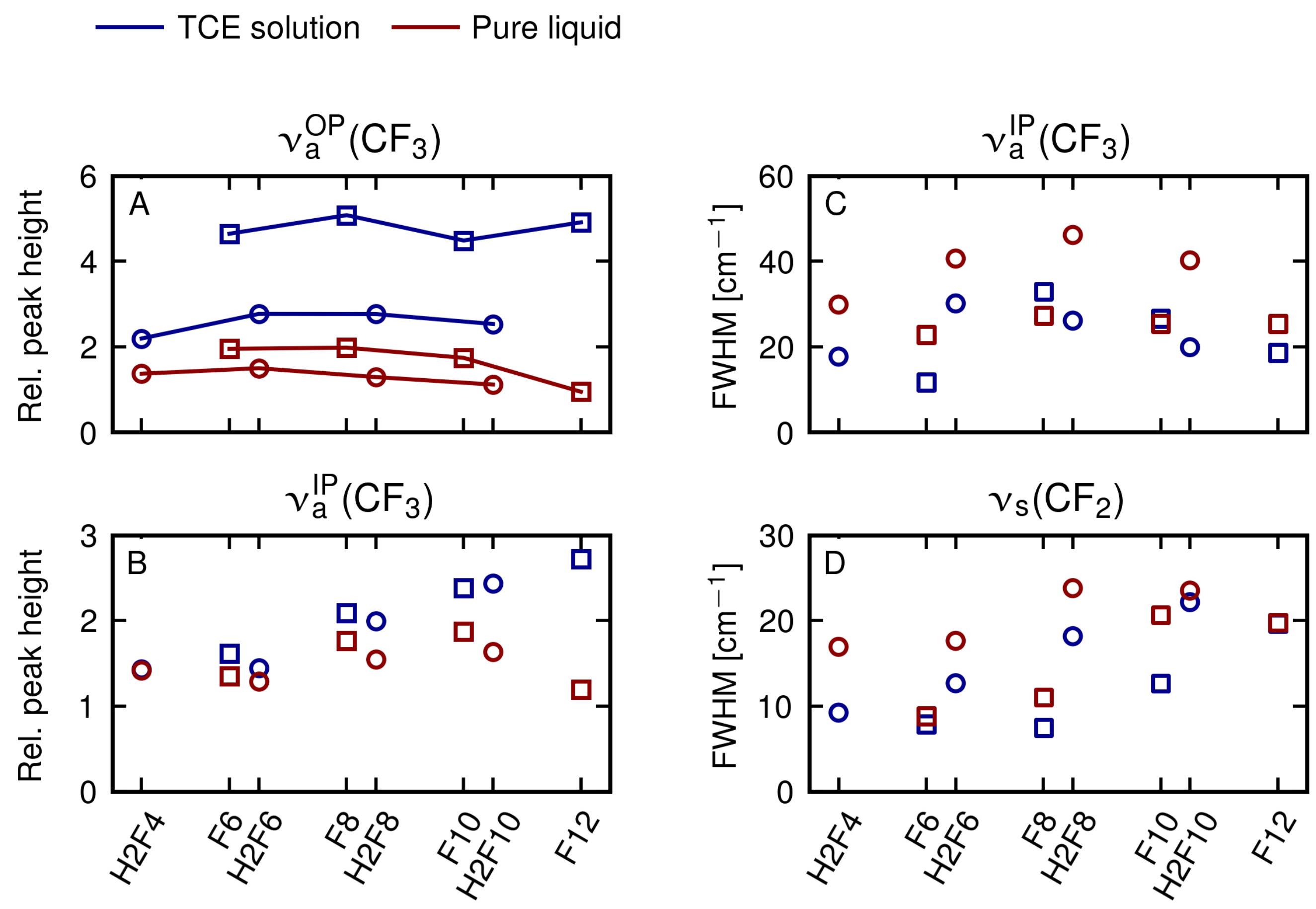

FIG. S11. **A**-**B**: Comparison of maximum peak heights of $\nu_a^{OP}(CF_3)$ (A) and $\nu_a^{IP}(CF_3)$ (B) bands normalized to the maximum height of $\nu_s(CF_2)$ peak for different molecular lengths. **C**-**D**: Peak widths of the $\nu_a^{IP}(CF_3)$ (C) and $\nu_s(CF_2)$ (D) vibrational bands for molecules of different lengths.

S7. COMPARISON OF DIFFERENT XC FUNCTIONALS AND HYDROGENATED CLUSTERS

All DFT calculations presented in the main text have been performed at the B97M-V/def2-TZVP level of theory using the ORCA/5.0.4 software suite. We employ default DFT integration grids, which yield a representation of the total electronic charge within 0.001 elementary charges of the expected total electronic charge. Self-Consistent-Field calculations are converged to a precision of 1.0e-8 Ha. For geometry optimizations, default geometry convergence criteria are employed which correspond to a maximum residual force of 1.0e-4 Bohr/Hartree and a maximum displacement of 4.0e-3 Bohr. Geometry convergence was verified by checking for the absence of any negative frequencies from the vibrational analysis.

To asses the influence of the exchange correlation functional, we compare vibrational spectra of single molecules as well as clusters of F2F4 and F6 at B3LYP-D3/6-31G(d,p) and MO6-2X-D3/6-31G(d,p) levels of theory in Fig. S12. These calculations have been performed in Gaussian 16.[5] We employ tight SCF convergence, corresponding to a convergence of energy to a precision of 1e-8 Ha as well as default geometry convergence criteria corresponding to a maximum residual force of 4.5e-4 Ha / Bohr and a maximum atomic displacement of 1.8e-3 Bohr.

Intensity decreases of the $\nu_a^{OP}(CF_3)$ and $\nu_a^{IP}(CF_3)$ modes when going from single molecules surrounded by implicit TCE (red lines) to fully flexible clusters (light blue lines) are found consistently for all functionals. The intensity of the $\nu_s(CF_2)$ mode, which we use for normalization in experimental spectra, remains largely unaffected for all functionals.

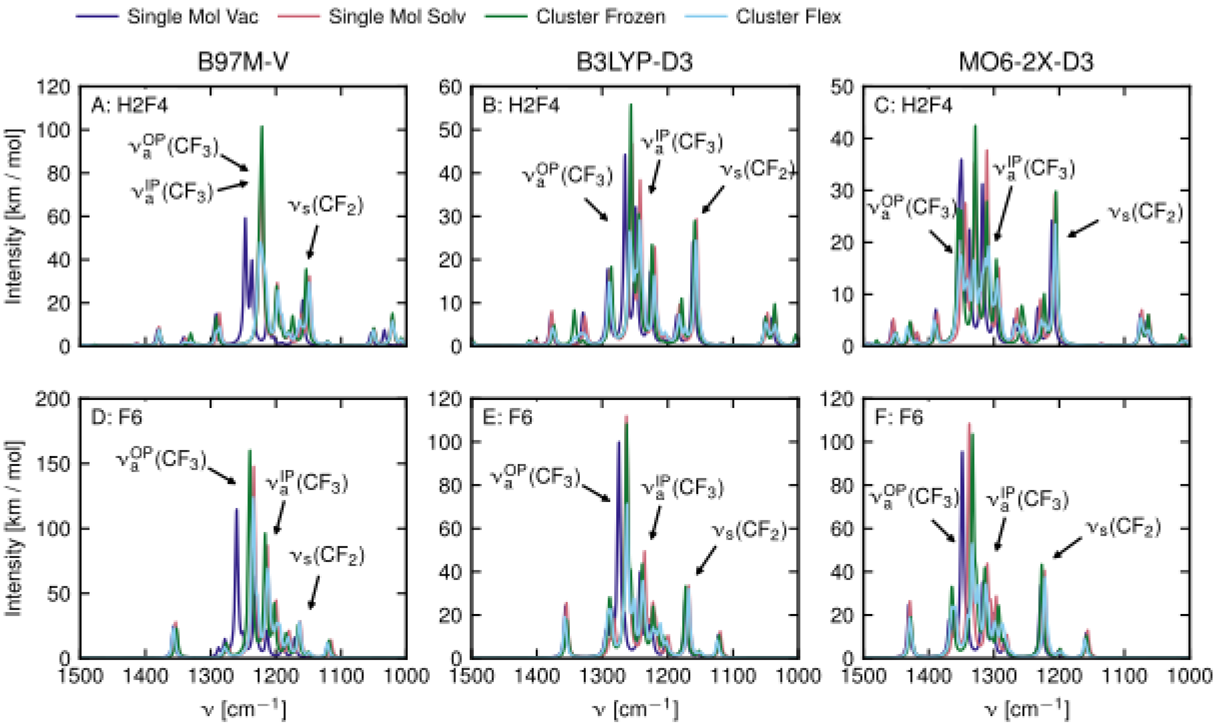

FIG. S12. Vibrational analysis of single molecules as well as clusters of H2F4 (**A** - **C**) and F6 (**D** - **F**) compounds for B97M-V/def2-TZVP, B3LYP-D3/6-31G(d,p) and MO6-2X-D3/6-31G(d,p) levels of theory.

To further verify that spectral changes observed in experiments as well as in DFT calculations reported in the main text originate from intramolecular vibrational coupling that is specific to fluorine-fluorine interactions, we compare DFT-based vibrational analysis of clusters of H2F4 (Fig. S13 A) and F6 (Fig. S13 B) molecules as shown in Fig. 5 of the main text with clusters of single H2F4 or F6 molecules surrounded by hexane molecules (see inset in panel B). For (partially) fluorinated clusters we find drops of intensity of the $\nu_a^{OP}(CF_3)$ and $\nu_a^{IP}(CF_3)$ modes when going from frozen (green lines) to flexible (light blue lines) clusters. For hydrogenated clusters on the other hand, only a small drop in intensity is found in case for H2F4 (red lines to yellow lines) and no effect is seen for F6, consistent with experimental data gathered from H2F4 and F6 molecules in various solvents.

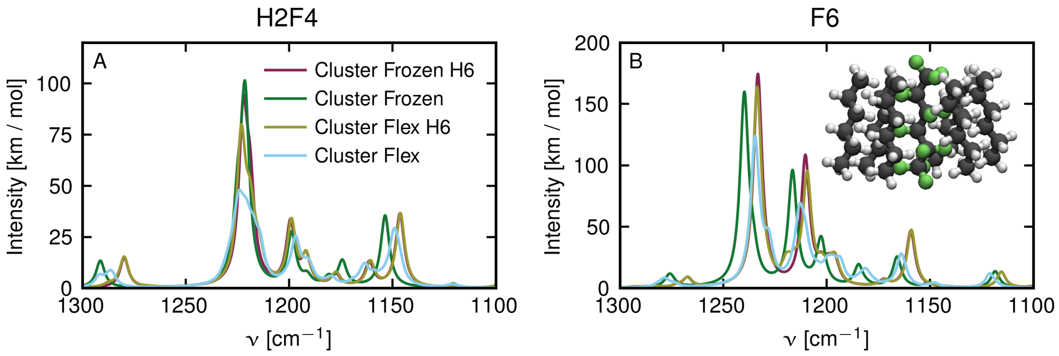

FIG. S13. Vibrational spectra of H2F4 (**A**) and F6 (**B**) molecules surrounded by fluorinated and hydrogenated clusters from static DFT calculations at the B97M-V/def2-TZVP level of theory. We compare vibrational spectra from frozen and flexible clusters.

S8. DECOMPOSITION OF FF-MD SPECTRA INTO SINGLE MOLECULAR AND COLLECTIVE CONTRIBUTIONS

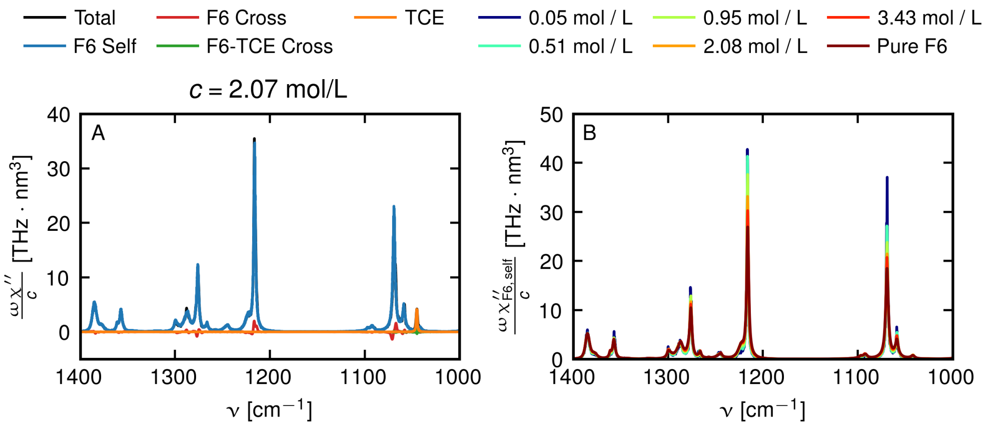

FIG. S14. **A**: Decomposition of the absorption spectrum of F6-TCE mixture at c(F6)= 2.08 mol/L from FF-MD simulations into different contributions according to Eq. S17. **B**: Self spectrum of F6 at various concentrations of F6-TCE mixtures.

In order to distinguish whether changes in the absorption spectrum in F6-TCE mixtures with changing F6 concentration are caused by the change of molecular motion or by collective polarization effects, we decompose the system dipole moment into molecular dipole moments $\boldsymbol{\mu}_i^{\mathrm{F6}}$ of F6 and $\boldsymbol{\mu}_i^{\mathrm{TCE}}$ of TCE molecules:

$$\mathbf{M}(t) = \sum_i^{N_{\mathrm{F6}}} \boldsymbol{\mu}_i^{\mathrm{F6}} + \sum_j^{N_{\mathrm{TCE}}} \boldsymbol{\mu}_j^{\mathrm{TCE}}. \tag{S16}$$

The dipole moment autocorrelation function, from which we calculate the absorption spectrum using Eq. 1 of the main text is then decomposed into

$$\begin{aligned}\langle \mathbf{M}(0)\cdot\mathbf{M}(t)\rangle =& \underbrace{\sum_i^{N_{\mathrm{F6}}} \left\langle \boldsymbol{\mu}_i^{\mathrm{F6}}(0)\cdot\boldsymbol{\mu}_i^{\mathrm{F6}}(t)\right\rangle}_{=\Phi_{\mathrm{F6,self}}(t)} + \underbrace{\sum_{i\neq j}^{N_{\mathrm{F6}}} \left\langle \boldsymbol{\mu}_i^{\mathrm{F6}}(0)\cdot\boldsymbol{\mu}_j^{\mathrm{F6}}(t)\right\rangle}_{=\Phi_{\mathrm{F6,cross}}(t)} + \underbrace{\sum_i^{N_{\mathrm{F6}}}\sum_j^{N_{\mathrm{TCE}}} \left[\left\langle \boldsymbol{\mu}_i^{\mathrm{F6}}(0)\cdot\boldsymbol{\mu}_j^{\mathrm{TCE}}(t)\right\rangle + \left\langle \boldsymbol{\mu}_j^{\mathrm{TCE}}(0)\cdot\boldsymbol{\mu}_i^{\mathrm{F6}}(t)\right\rangle\right]}_{=\Phi_{\mathrm{F6,TCE}}(t)} \\ &+ \underbrace{\sum_i^{N_{\mathrm{TCE}}}\sum_j^{N_{\mathrm{TCE}}} \left\langle \boldsymbol{\mu}_i^{\mathrm{TCE}}(0)\boldsymbol{\mu}_j^{\mathrm{TCE}}(t)\right\rangle}_{=\Phi_{\mathrm{TCE}}(t)}.\end{aligned} \tag{S17}$$

The contributions to the absorption spectrum are defined accordingly. In Fig. S14 A we depict the different contributions for a concentration of $c = 2.08$ mol/L of F6 in TCE. In the displayed frequency regime, and in particular for the three modes discussed in the main text, $\nu_a^{IP}(CF_3)$, $\nu_a(CF_2)$ and $\nu_s(CF_2)$, the self-contribution of F6 molecules (blue line) dominates. F6 cross-correlations (red line) give minor contributions while no significant polarization correlations are found between F6 and TCE (green line). The 1045 cm^{-1} peak is found to be caused by TCE vibrations (orange line), as we already argued in the main text based on the concentration dependence of the spectra.

Since the overall spectrum (black line) is nearly entirely accounted for by individual F6 vibrations, the strong concentration dependence of the $\nu_a^{IP}(CF_3)$, $\nu_a(CF_2)$ and $\nu_s(CF_2)$ modes arises from a change in the molecular motion of F6 molecules. Direct evidence for this is presented in Fig. S14 B where we show the F6 self-spectrum as a function of concentration. This spectrum reproduces the decreases in maximum peak height observed in Fig. 6 of the main text.

S9. FF-MD SPECTRA OF MOLECULES WITH AND WITHOUT GAUCHE DEFFECTS

The projection of molecular motion onto individual normal modes outlined in the main text is based solely on the analysis of trans conformers. To make sure that the spectral changes we observe are not actually caused by gauche conformers, we decompose the FF-MD spectra of F2F4/TCE mixtures shown in Fig. 6 into contributions trans gauche conformers. To do so, gauche conformers are identified based on the criterium that at least one of the backbone dihedral angles takes a value larger than 90°. Trajectories were divided into all-trans and all-gauche subtrajectories and over those trajectories vibrational self spectra were determined according to Eq. S17. The resulting spectra (Fig. S15) reveal that gauche conformers gives contributions at frequencies slightly shifted with respect to the main absorption features. The spectra of trans conformers on the other hand reproduce the line broadening discussed in the main text.

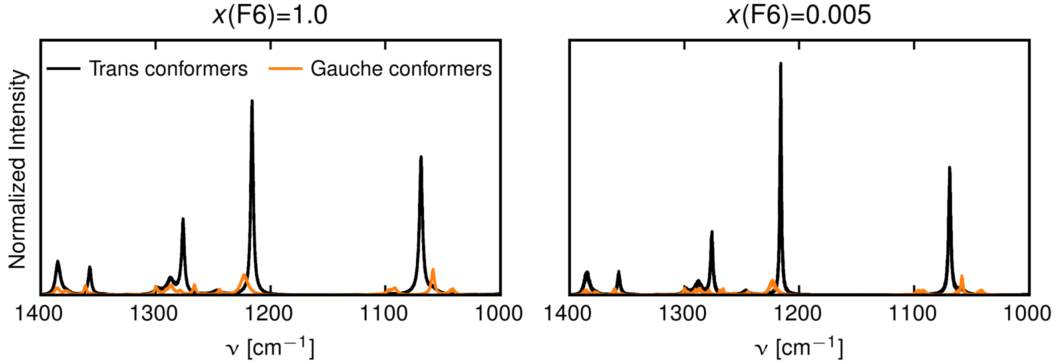

FIG. S15. Spectra of all-trans conformers (black line) and gauche conformers from FF-MD simulation of bulk diluted F6 in TCE and pure F6. Relative population of all-trans to gauche conformers is approximately 9:1 fr both concentrations.

S10. EXPRESSING THE ENERGY ABSORPTION SPECTRUM IN TERMS OF VIBRATIONAL NORMAL MODES

The configuration of a molecule can be described by the configuration vector $\mathbf{x}(t)$ containing $3N$ Cartesian coordinates, where N is the number of atoms in the molecule. For each time step, we determine whether the backbone helix of the molecule is left or right-handed and describe this with the indicator function

$$\theta(t) = \begin{cases} 1 & \text{if} \quad \phi_{C_1-C_2-C_5-C_6}(t) > 0\,, \\ 0 & \text{if} \quad \phi_{C_1-C_2-C_5-C_6}(t) < 0\,. \end{cases} \tag{S18}$$

For the two different helicities, we perform separate geometry optimizations employing the same force-field as was used for the FF-MD simulations. This results in the reference structures $\mathbf{x}_{ref}^{+}$ and $\mathbf{x}_{ref}^{-}$, for right-handed and left-handed helicity, respectively. Each molecular structure $\mathbf{x}(t)$ from the MD simulations is fitted onto the reference struture at corresponding helicity, by minimizing the mean square distance between the carbon atoms using algorithms implemented in the MDAnalysis software package.[6–8] This fit gives a rotation matrix $\mathbf{R}(t)$ and a translation vector $\mathbf{x}_0(t)$ such that the fitted configuration described by

$$\mathbf{x}_{\text{fit}}(t) = \mathbf{R}(t)\,[\mathbf{x}(t) - \mathbf{x}_0(t)] \tag{S19}$$

is optimally aligned with the corresponding reference structure. We perform normal mode analysis of the two reference structures resulting into two sets of $3N$ normal mode vectors $\boldsymbol{\eta}_k^+$ and $\boldsymbol{\eta}_k^-$ ($k \in [1, ..., 3N]$). By projecting the fitted configuration vector $\mathbf{x}_{\text{fit}}$ onto the normal mode vectors, again taking into account the backbone helicity, we find a new set of coordinates $\mathbf{q}(t)$, whose $3N$ components are given by

$$\begin{aligned} q_k(t) &= \theta(t)\left(\mathbf{x}_{\text{fit}}(t) - \mathbf{x}_{\text{ref}}^+\right) \cdot \boldsymbol{\eta}_k^+ + (1-\theta(t))\left(\mathbf{x}_{\text{fit}}(t) - \mathbf{x}_{\text{ref}}^-\right) \cdot \boldsymbol{\eta}_k^- \\ &= \left(\mathbf{x}_{\text{fit}}(t) - \mathbf{x}_{\text{ref}}^-\right) \cdot \boldsymbol{\eta}_k^- + \theta(t)\left[\left(\mathbf{x}_{\text{fit}}(t) - \mathbf{x}_{\text{ref}}^+\right) \cdot \boldsymbol{\eta}_k^+ - \left(\mathbf{x}_{\text{fit}}(t) - \mathbf{x}_{\text{ref}}^-\right) \cdot \boldsymbol{\eta}_k^-\right] . \end{aligned} \tag{S20}$$

They describe the molecular configuration with respect to the normal mode basis. Note, that $\theta(t)$ is not identical to the helicity indicator function θ_{hel} in the main text. In order to derive an exact relation with the polarization, we cannot not discard gauche conformers. The first six components of $\mathbf{q}$, which correspond to the zero frequency normal modes associated with rotations and translations, are zero, since we already determined those independently in Eq. S19. We can express the configuration vector in the basis of normal mode vectors as

$$\begin{aligned} \mathbf{x}_{\text{fit}}(t) &= \theta(t)\left[\sum_k q_k(t)\boldsymbol{\eta}_k^+ + \mathbf{x}_{\text{ref}}^+\right] + (1-\theta(t))\left[\sum_k q_k(t)\boldsymbol{\eta}_k^- + \mathbf{x}_{\text{ref}}^-\right] \\ &= \sum_k q_k(t)\boldsymbol{\eta}_k^- + \mathbf{x}_{\text{ref}}^- + \theta(t)\left[\sum_k q_k(t)\left(\boldsymbol{\eta}_k^+ - \boldsymbol{\eta}_k^-\right) + \mathbf{x}_{\text{ref}}^+ - \mathbf{x}_{\text{ref}}^-\right] . \end{aligned} \tag{S21}$$

Within the framework of the decomposition in Eq. S17 we can express the self part of the F6 dipole moment autocorrelation function in terms of the normal mode projection q_k. All collective parts cannot be treated within the normal mode analysis formalism and are discarded here. The F6 self part of the spectrum, *i.e.* the dipole autocorrelation function of a molecule's dipole moment, is given by

$$\langle \boldsymbol{\mu}(0) \cdot \boldsymbol{\mu}(t) \rangle = \langle \mathbf{Q}\mathbf{x}(0) \cdot \mathbf{Q}\mathbf{x}(t) \rangle , \tag{S22}$$

where $\mathbf{Q}$ denotes a $3 \times 3N$ matrix containing the partial charges of all the atoms. Rearranging Eq. S19 to

$$\mathbf{x}(t) = \mathbf{R}^{-1}(t)\mathbf{x}_{\text{fit}}(t) + \mathbf{x}_0(t) \tag{S23}$$

and using Eq. S21, we can express $\boldsymbol{\mu}(t)$ as:

$$\boldsymbol{\mu}(t) = \mathbf{Q}\mathbf{R}^{-1}(t)\left(\sum_k q_k(t)\boldsymbol{\eta}_k^- + \mathbf{x}_{\text{ref}}^- + \theta(t)\left[\sum_k q_k(t)(\boldsymbol{\eta}_k^+ - \boldsymbol{\eta}_k^-) + \mathbf{x}_{\text{ref}}^+ - \mathbf{x}_{\text{ref}}^-\right]\right) + \mathbf{Q}\mathbf{x}_0(t) . \tag{S24}$$

In case of neutral molecules, the rigid body translation $\mathbf{x}_0(t)$ does not cause a change of dipole movement, *i.e.*

$$\mathbf{Q}\mathbf{x}_0(t) = \mathbf{0} . \tag{S25}$$

Note that $\mathbf{Q}$ and $\mathbf{R}^{-1}$ do not commute. In order to apply the charge matrix onto the configuration vector prior to the rotation, we can however define a new rotation matrix

$$\tilde{\mathbf{R}}^{-1}(t) = \mathbf{Q}\mathbf{R}^{-1}(t)\mathbf{Q}^{-1} \tag{S26}$$

with $\mathbf{Q}^{-1}$ being the right inverse of $\mathbf{Q}$ ($\mathbf{Q}$ is not a square matrix and thus not generally invertible) such that

$$\mathbf{Q}\mathbf{R}^{-1}(t) = \tilde{\mathbf{R}}^{-1}(t)\mathbf{Q} . \tag{S27}$$

Like this, $\tilde{\mathbf{R}}^{-1}(t)$ is the matrix, which describes the rotation of the dipole moment of a molecule with configuration vector $\mathbf{x}$ and partial charges $\mathbf{Q}$. Applying the charge matrix to the configuration terms in Eq. S24, we find terms of the type

$$\mathbf{Q}\left[\sum_k q_k(t)\boldsymbol{\eta}_k^+ + \mathbf{x}_{\text{ref}}^+\right] = \sum_k q_k(t)\boldsymbol{\mu}_k^+ + \boldsymbol{\mu}_{\text{ref}}^+ , \tag{S28}$$

where $\boldsymbol{\mu}_{\text{ref}}^+$ denotes the dipole moment of the molecule in the $(+)$-reference configuration and $\boldsymbol{\mu}_k^+$ denotes the transition dipole moment of the k-th normal mode in the $(+)$-reference configuration. Corresponding quantities are also defined for the $(-)$-reference structures. We can thus write the total dipole moment trajectory in terms of a vibrational (vib) and a rigid body (rb) component using Eq. 5 of the main text:

$$\boldsymbol{\mu}(t) = \boldsymbol{\mu}^{\text{rb}}(t) + \sum_k \boldsymbol{\mu}_k^{\text{vib}}(t) , \tag{S29}$$

with

$$\boldsymbol{\mu}^{\mathrm{rb}}(t) = \tilde{\mathbf{R}}^{-1}(t)\boldsymbol{\mu}_{\mathrm{ref}}^{-} + \theta(t)\tilde{\mathbf{R}}^{-1}(t)\left(\boldsymbol{\mu}_{\mathrm{ref}}^{+} - \boldsymbol{\mu}_{\mathrm{ref}}^{-}\right), \tag{S30}$$

$$\boldsymbol{\mu}_k^{\mathrm{vib}}(t) = \tilde{\mathbf{R}}^{-1}(t)q_k(t)\boldsymbol{\mu}_k^{-} + \theta(t)\tilde{\mathbf{R}}^{-1}(t)q_k(t)(\boldsymbol{\mu}_k^{+} - \boldsymbol{\mu}_k^{-}). \tag{S31}$$

Evaluating Eq. S22 we find

$$\langle \boldsymbol{\mu}(0)\cdot\boldsymbol{\mu}(t)\rangle = \left\langle \sum_k \boldsymbol{\mu}_k^{\mathrm{vib}}(0)\cdot\sum_k \boldsymbol{\mu}_k^{\mathrm{vib}}(t)\right\rangle + 2\left\langle \boldsymbol{\mu}^{\mathrm{rb}}(0)\cdot\sum_k \boldsymbol{\mu}_k^{\mathrm{vib}}(t)\right\rangle + \left\langle \boldsymbol{\mu}^{\mathrm{rb}}(0)\cdot\boldsymbol{\mu}^{\mathrm{rb}}(t)\right\rangle. \tag{S32}$$

This can be fully expanded into:

$$\langle \boldsymbol{\mu}(0)\cdot\boldsymbol{\mu}(t)\rangle =$$

$$\left\langle \tilde{\mathbf{R}}^{-1}(0)\sum_k q_k(0)\boldsymbol{\mu}_k^{-}\cdot\tilde{\mathbf{R}}^{-1}(t)\sum_k q_k(t)\boldsymbol{\mu}_k^{-}\right\rangle \tag{S33a}$$

$$+\left\langle \tilde{\mathbf{R}}^{-1}\theta(0)\sum_k q_k(0)(\boldsymbol{\mu}_k^{+}-\boldsymbol{\mu}_k^{-})\cdot\tilde{\mathbf{R}}^{-1}\theta(t)\sum_k q_k(t)(\boldsymbol{\mu}_k^{+}-\boldsymbol{\mu}_k^{-})\right\rangle \tag{S33b}$$

$$+2\left\langle \tilde{\mathbf{R}}^{-1}\sum_k q_k(0)\boldsymbol{\mu}_k^{-}\cdot\tilde{\mathbf{R}}^{-1}(t)\theta(t)\sum_k q_k(t)(\boldsymbol{\mu}_k^{+}-\boldsymbol{\mu}_k^{-})\right\rangle \tag{S33c}$$

$$+2\left\langle \tilde{\mathbf{R}}^{-1}(0)\boldsymbol{\mu}_{\mathrm{ref}}^{-}\cdot\tilde{\mathbf{R}}^{-1}(t)\sum_k q_k(t)\boldsymbol{\mu}_k^{-}\right\rangle \tag{S33d}$$

$$+2\left\langle \tilde{\mathbf{R}}^{-1}(0)\theta(0)(\boldsymbol{\mu}_{\mathrm{ref}}^{+}-\boldsymbol{\mu}_{\mathrm{ref}}^{-})\cdot\tilde{\mathbf{R}}^{-1}(t)\theta(t)\sum_k q_k(t)(\boldsymbol{\mu}_k^{+}-\boldsymbol{\mu}_k^{-})\right\rangle \tag{S33e}$$

$$+2\left\langle \tilde{\mathbf{R}}^{-1}(0)\sum_k q_k(0)\boldsymbol{\mu}_k^{-}\cdot\tilde{\mathbf{R}}^{-1}(t)t\theta(t)(\boldsymbol{\mu}_{\mathrm{ref}}^{+}-\boldsymbol{\mu}_{\mathrm{ref}}^{-})\right\rangle \tag{S33f}$$

$$+2\left\langle \tilde{\mathbf{R}}^{-1}(0)\boldsymbol{\mu}_{\mathrm{ref}}^{-}\cdot\tilde{\mathbf{R}}^{-1}(t)\theta(t)\sum_k q_k(t)(\boldsymbol{\mu}_k^{+}-\boldsymbol{\mu}_k^{-})\right\rangle \tag{S33g}$$

$$+\langle \tilde{\mathbf{R}}^{-1}(0)\boldsymbol{\mu}_{\mathrm{ref}}^{-}\cdot\tilde{\mathbf{R}}^{-1}(t)\boldsymbol{\mu}_{\mathrm{ref}}^{-}\rangle \tag{S33h}$$

$$+\langle \tilde{\mathbf{R}}^{-1}(0)\theta(0)(\boldsymbol{\mu}_{\mathrm{ref}}^{+}-\boldsymbol{\mu}_{\mathrm{ref}}^{-})\cdot\tilde{\mathbf{R}}^{-1}(t)\theta(t)(\boldsymbol{\mu}_{\mathrm{ref}}^{+}-\boldsymbol{\mu}_{\mathrm{ref}}^{-})\rangle \tag{S33i}$$

$$+2\langle \tilde{\mathbf{R}}^{-1}(0)\boldsymbol{\mu}_{\mathrm{ref}}^{-}\cdot\tilde{\mathbf{R}}^{-1}(t)\theta(t)(\boldsymbol{\mu}_{\mathrm{ref}}^{+}-\boldsymbol{\mu}_{\mathrm{ref}}^{-})\rangle\,. \tag{S33j}$$

Rigid body rotations are slow compared to intermolecular vibrations. The F6 absorption spectrum in the frequency regime we are interested in (1000 - 1400 cm^{-1}), can be accurately described by the vibrational part (1st term in Eq. S32, corresponding to Eqs. S23a - S23c), as is shown in Fig. 7 F of the main text. To investigate the influence of helicity reversal on the absorption spectrum, we assume that left- and right-handed conformations have identical transition dipole moments, *i.e.* $\boldsymbol{\mu}_k^{+} = \boldsymbol{\mu}_k^{-}$. We further assume, that molecular rotations are slow in time, such that rigid body rotation can be expressed as $\tilde{\mathbf{R}}^{-1}(t) = \tilde{\mathbf{R}}^{-1}(0) + \delta t\,\frac{\partial \tilde{\mathbf{R}}^{-1}(t)}{\partial t}\Big|_{t=0} + \mathscr{O}(\delta^2 t^2)$. To zeroth order in the slowly varying time δt, $\delta \ll 1$, we find

$$\left\langle \boldsymbol{\mu}^{\mathrm{vib}}(0)\cdot\boldsymbol{\mu}^{\mathrm{vib}}(t)\right\rangle = \left\langle \sum_k \boldsymbol{\mu}_k^{\mathrm{vib}}(0)\cdot\sum_k \boldsymbol{\mu}_k^{\mathrm{vib}}(t)\right\rangle = \sum_k |\boldsymbol{\mu}_k^{-}|^2\,\langle q_k(0)q_k(t)\rangle + \mathscr{O}(\delta), \tag{S34}$$

where we used the fact that normal modes are mutually orthogonal $\boldsymbol{\mu}_i^{-}\cdot\boldsymbol{\mu}_j^{-} = \delta_{ij}$. The power spectra corresponding to the $\langle q_k(0)q_k(t)\rangle$ autocorrelation function are shown in Fig. 7 H of the main text.

S11. DIHEDRAL HELICITY - TRANSITION STATE THEORY

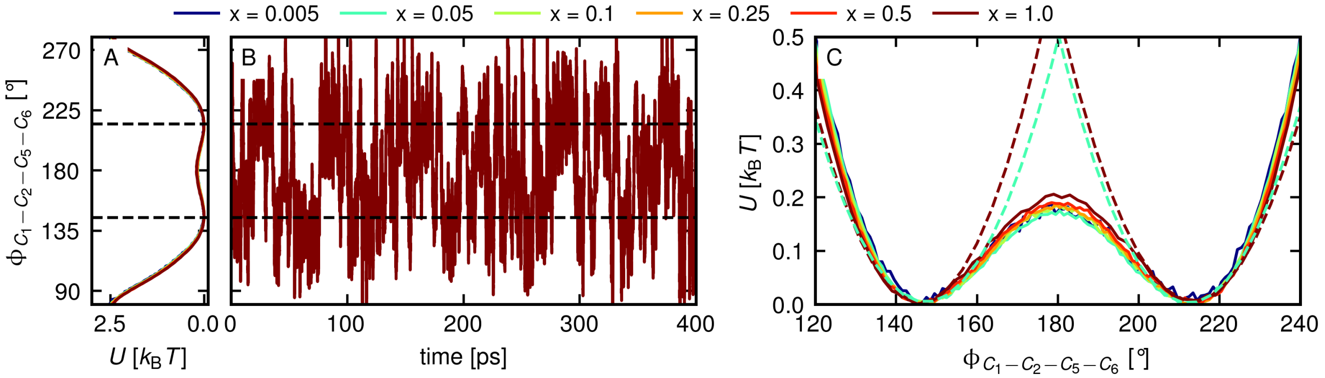

FIG. S16. **A**: Free energy profile of the helicity dihedral angle $\phi_{C_1-C_2-C_5-C_6}$ of F6 for different concentrations of TCE. Minimum energy angles are indicated by black dashed lines. **B**: Representative trajectory of the dihedral angle for pure liquid F6. **C**: Close up of the energy barrier separating left- and right-turning helices. Quadratic fits of the energy minima are indicated as dashed lines for selected concentrations.

In order to estimate, whether the speed-up of helix reversal can be explained by changes in the free energy profile, we compare the simulated mean helix reversal relaxation time to predictions from transition state theory. According to transition state theory, which is valid in the intermediate friction regime,[9] the characteristic time for a barrier-crossing event is given as[10]

$$\tau_\mathrm{TST} = 2\pi\sqrt{\frac{m}{\Delta U''_\mathrm{min}}}e^{\beta\Delta U}\,, \tag{S35}$$

where β is the inverse thermal energy, ΔU refers to the barrier height, $\Delta U''_\mathrm{min}$ to the curvature of the free energy at the energy minimum, and m refers to the mass of the reaction coordinate. We estimate the ratio of the transition time for helix reversal for diluted F6 in TCE (x=0.05) and pure F6 by fitting the free energy profile of the overall helix dihedral $\phi_{C_1-C_2-C_5-C_6}$ with a second order polynomial in the vicinity of the energy minima to find the curvature and by extracting the barrier height; see Fig. S16. Assuming that the mass of the reaction coordinate remains invariant when the chemical environment changes, which is an approximation,[11] the ratio is given by

$$\frac{\tau^\mathrm{dilute}_\mathrm{TST}}{\tau^\mathrm{pure}_\mathrm{TST}} = e^{\beta(\Delta U^\mathrm{dilute}-\Delta U^\mathrm{pure})}\sqrt{\frac{\Delta U''^\mathrm{pure}_\mathrm{min}}{\Delta U''^\mathrm{dilute}_\mathrm{min}}}\,. \tag{S36}$$

We find barrier heights of $\Delta U^\mathrm{dilute} = 0.18\ k_\mathrm{B}T$ for the x=0.05 and $\Delta U^\mathrm{pure} = 0.205\ k_\mathrm{B}T$ of for the pure system, and curvatures in the free energy minimum of $\Delta U''^\mathrm{dilute}_\mathrm{min} = 4.557\cdot 10^{-4} k_\mathrm{B}T/\mathrm{degree}^2$ and $\Delta U''^\mathrm{pure}_\mathrm{min} = 5.482\cdot 10^{-4} k_\mathrm{B}T/\mathrm{degree}^2$, respectively. This results in an estimated ratio of transition time of $\frac{\tau^\mathrm{dilute}_\mathrm{TST}}{\tau^\mathrm{pure}_\mathrm{TST}} \approx 1.07$, which does not account for the speed-up of helical reversal of nearly a factor of 2 reported in Fig. 7 D-E of the main text. A dissipative process induced by dynamical coupling of the F6 dihedral to the F6 environment is the most plausible mechanism to explain the discrepancy between transition state theory and FF-MD simulation results.[12]

S12. HELICITY OF SHORT PERFLUOROALKYL CHAINS

PFAS are known to assume a helical conformation for various chain lengths.[13] To determine the dependence of the backbone helicity on the number of perfluorinated carbon atoms in the alkylchain, we performed DFT geometry optimizations on the B3LYP/6-311G(d,p) level of theory employing the Gaussian/16 software suits. Convergence criteria etc. are chosen the same way as detailed in Sec. S7. We find that for both types of considered compounds F(N) and H2F(N), the minimum energy structure is helical for all $N \geq 4$. The shortest helical compounds H2F4 and F4 are shown in Fig. S17. At a lower number of perfluorinated carbon atoms, for example for the H2F3 compound or the F3 compound, the minimum energy geometry remains flat.

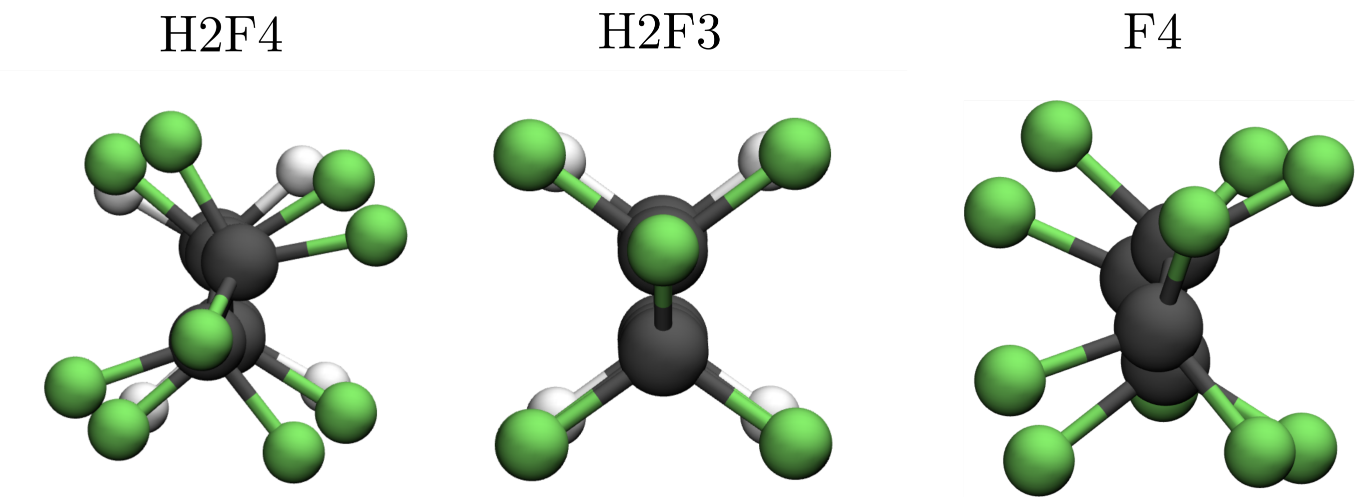

FIG. S17. Optimized geometries of the H2F3, H2F4 and F4 compounds from DFT calculations on the B3LYP/6-311G(d,p) level of theory.

In the main text, we show how the lifetime of the backbone helicity of F6 molecules influences the line shape of $\nu(CF_n)$ bands in F6/TCE solutions. In Fig. S18 we repeat this analysis for H2F4/TCE mixtures. We compare the highly diluted case (blue lines) with the pure H2F4 liquid. Similar to F6 spectra shown in Fig. 6 of the main text, a reduction in peak height is observed for the $\nu_a^{IP}(CF_3)$, $\nu_a(CF_2)$ and $\nu_s(CF_2)$ modes. We link this to a reduction of the helicity lifetime shown in Fig. S18 B. In the displayed helicity autocorrelation function, two exponential relaxation processes can be distinguished. The long relaxation time of the order of hundreds of ps corresponds to transitions between gauche and trans conformers which happens on the same timescale for the two displayed systems, while the short relaxation time corresponds to helicity reversal. Similar to F6, the helicity reversal time decreases for pure H2F4 compared to dilute H2F4/TCE from 1 ps to 0.65 ps.

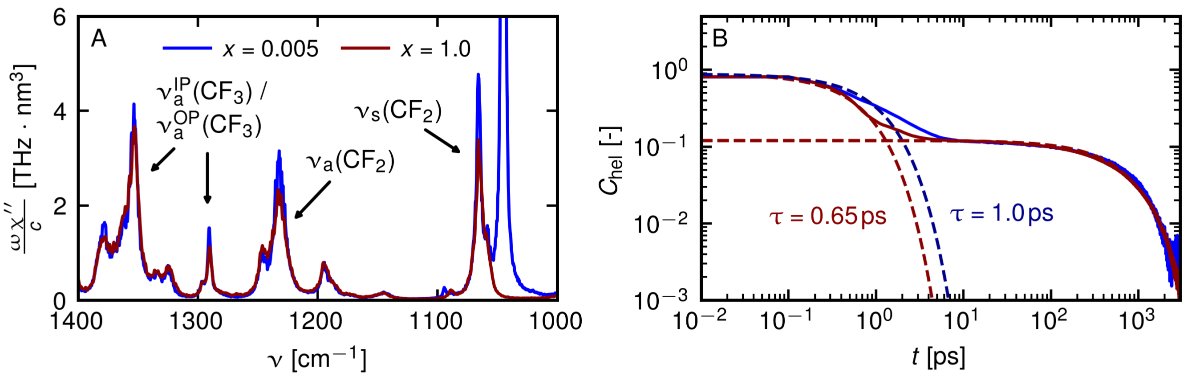

FIG. S18. **A**: FF-MD spectra of dilute H2F4/TCE solution (blue line) and pure bulk H2F4 liquid (red line). **B**: Helicity autocorrelation function using Eq. 7 of the main text fitted by two exponential functions $e^{-t\tau}$ per system (dashed lines).

S13. INFLUENCE OF HELICITY REVERSAL ON DIFFERENT SPECTRAL BANDS

[1] T. Hasegawa, *Quantitative Infrared Spectroscopy for Understanding of a Condensed Matter* (Springer Japan, 2017) pp. 1–200.
[2] T. Hasegawa, Physicochemical Nature of Perfluoroalkyl Compounds Induced by Fluorine, Chemical Record **17**, 903 (2017).
[3] J. S. Plaskett and P. N. Schatz, On the Robinson and Price (Kramers-Kronig) method of interpreting reflection data taken through a transparent window, The Journal of Chemical Physics **38**, 612 (1963).
[4] J. A. Bardwell and M. J. Dignam, Extensions of the Kramers-Kronig transformation that cover a wide range of practical spectroscopic applications, The Journal of Chemical Physics **83**, 5468 (1985).

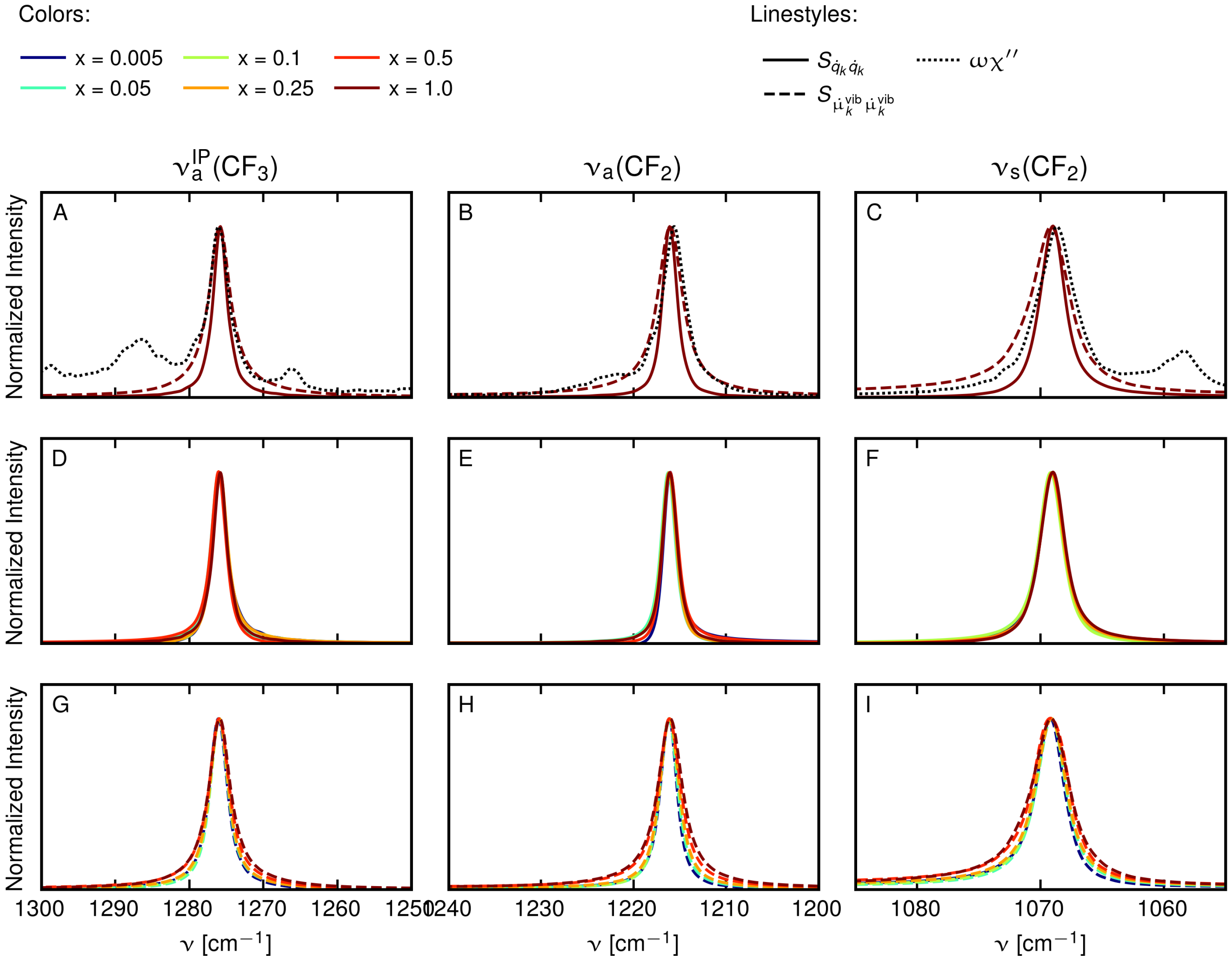

FIG. S19. Full vibrational dipole spectra (dashed lines) and vibrational dipole spectra of F6 containing no contribution from helicity reversal (solid lines) of the $\nu_a^{\mathrm{IP}}(CF_3)$, $\nu_a(CF_2)$ and ν_s(CF$_2$ modes compared to the normalized polarization based absorption spectrum $\omega\chi''$. The relation of the three different spectra is explained in the main text. Here, we note that for all displayed vibrational modes, $S_{\dot{q}_k \dot{q}_k}$ is independent of F6 concentration, while $S_{\dot{\boldsymbol{\mu}}_k^{\mathrm{vib}} \dot{\boldsymbol{\mu}}_k^{\mathrm{vib}}}$ shows line broadening with increasing concentration.

[5] M. J. Frisch, G. W. Trucks, H. B. Schlegel, G. E. Scuseria, M. A. Robb, J. R. Cheeseman, G. Scalmani, V. Barone, G. A. Petersson, H. Nakatsuji, X. Li, M. Caricato, A. V. Marenich, J. Bloino, B. G. Janesko, R. Gomperts, B. Mennucci, H. P. Hratchian, J. V. Ortiz, A. F. Izmaylov, J. L. Sonnenberg, D. Williams-Young, F. Ding, F. Lipparini, F. Egidi, J. Goings, B. Peng, A. Petrone, T. Henderson, D. Ranasinghe, V. G. Zakrzewski, J. Gao, N. Rega, G. Zheng, W. Liang, M. Hada, M. Ehara, K. Toyota, R. Fukuda, J. Hasegawa, M. Ishida, T. Nakajima, Y. Honda, O. Kitao, H. Nakai, T. Vreven, K. Throssell, J. A. Montgomery, Jr., J. E. Peralta, F. Ogliaro, M. J. Bearpark, J. J. Heyd, E. N. Brothers, K. N. Kudin, V. N. Staroverov, T. A. Keith, R. Kobayashi, J. Normand, K. Raghavachari, A. P. Rendell, J. C. Burant, S. S. Iyengar, J. Tomasi, M. Cossi, J. M. Millam, M. Klene, C. Adamo, R. Cammi, J. W. Ochterski, R. L. Martin, K. Morokuma, O. Farkas, J. B. Foresman, and D. J. Fox, Gaussian˜16 Revision C.01 (2016), gaussian Inc. Wallingford CT.

[6] D. L. Theobald, Rapid calculation of rmsds using a quaternion-based characteristic polynomial, Foundations of Crystallography **61**, 478 (2005).

[7] P. Liu, D. K. Agrafiotis, and D. L. Theobald, Fast determination of the optimal rotational matrix for macromolecular superpositions, Journal of computational chemistry **31**, 1561 (2010).

[8] R. J. Gowers, M. Linke, J. Barnoud, T. J. E. Reddy, M. N. Melo, S. L. Seyler, J. Domanski, D. L. Dotson, S. Buchoux, I. M. Kenney, *et al.*, *Proceedings of the 15th Python in Science Conference*, Tech. Rep. (Los Alamos National Laboratory (LANL), Los Alamos, NM (United States), 2019).

[9] F. N. Brunig, J. O. Daldrop, and R. R. Netz, Pair-reaction dynamics in water: competition of memory, potential shape, and inertial effects, The Journal of Physical Chemistry B **126**, 10295 (2022).

[10] D. Chandler, Statistical mechanics of isomerization dynamics in liquids and the transition state approximation, The Journal of Chemical

Physics **68**, 2959 (1978).

[11] C. Ayaz, L. Scalfi, B. A. Dalton, and R. R. Netz, Generalized langevin equation with a nonlinear potential of mean force and nonlinear memory friction from a hybrid projection scheme, Physical review E **105**, 054138 (2022).

[12] J. Kappler, J. O. Daldrop, F. N. Brünig, M. D. Boehle, and R. R. Netz, Memory-induced acceleration and slowdown of barrier crossing, The Journal of Chemical Physics **148** (2018).

[13] S. S. Jang, M. Blanco, W. A. Goddard, G. Caldwell, and R. B. Ross, The source of helicity in perfluorinated n-alkanes, Macromolecules **36**, 5331 (2003).
